# A New Record of *Auricularia heimuer* in Thailand: Optimal Conditions for Mycelial Growth, Inoculum Production, Cultivation Performance and a Favorable Nutritional Profile

**DOI:** 10.3390/life16101647

**Published:** 2026-09-30

**Authors:** Kritsana Jatuwong, Jutamart Monkai, Worawoot Aiduang, Orlavanh Xayyavong, Phongeun Sysouphanthong, Didsanutda Gonkhom, Alviti Kankanamalage Hasith Priyashantha, Shihui Wang, Saisamorn Lumyong

**Affiliations:** 1 Office of Research Administration, Chiang Mai University, Chiang Mai 50200, Thailand; kritsana.ja@cmu.ac.th (K.J.); jutamart.mon@cmu.ac.th (J.M.); worawoot.aiduang@cmu.ac.th (W.A.); 2 Department of Biology, Faculty of Science, Chiang Mai University, Chiang Mai 50200, Thailand; xorlavanh@yahoo.com (O.X.); laofungi@gmail.com (P.S.); didsanutda8725@gmail.com (D.G.); priyashanthahasith@gmail.com (A.K.H.P.); wangshihui.mocha@gmail.com (S.W.); 3 Department of Biology, Faculty of Science, Champasack University, Pakse 16010, Laos; 4 Center of Excellence in Microbial Diversity and Sustainable Utilization, Chiang Mai University, Chiang Mai 50200, Thailand; 5 Academy of Science, The Royal Society of Thailand, Bangkok 10300, Thailand

**Keywords:** *Auricularia heimuer*, first record, mycelial growth optimization, mushroom cultivation, taxonomy, sustainable waste utilization

## Abstract

*Auricularia heimuer*, an economically important edible mushroom species, is reported for the first time in Thailand based on combined morphological and molecular analyses. Three strains (SL115, SL150, and SL160) were collected from decaying wood in dipterocarp forests in Phayao and Lampang provinces, northern Thailand. Phylogenetic analyses based on combined ITS and nLSU sequence data confirmed their identity as *A. heimuer*, with morphological characteristics consistent with the type description. Mycelial growth experiments demonstrated that culture medium, temperature, pH, and nitrogen source significantly influenced mycelial growth and biomass production. Potato dextrose agar (PDA) was the most suitable medium for strains SL115 and SL150, whereas corn dust agar (CDA) was optimal for SL160. All strains exhibited optimal growth at 30 °C under acidic to neutral pH conditions and in media supplemented with organic nitrogen sources, particularly tryptone, peptone, and yeast extract. For inoculum production, potato dextrose broth and corn stalk-based media were the most suitable substrates for liquid spawn, while corn kernels and barley were optimal for grain spawn, rubber sawdust for solid spawn, and lotus stalk, giant mimosa stalk, and rubber tree wood for stick spawn. Cultivation trials showed that *A. heimuer* strains SL115 and SL150 produced lower yield and biological efficiency (BE) than *A. cornea* SL167 when grown on a 100% rubber sawdust substrate. However, supplementation of the cultivation substrate with *Dipterocarpus* or teak leaves significantly improved productivity. Among the tested treatments, T10 (*A. heimuer* SL150 cultivated on a substrate containing 50% rubber sawdust and 50% *Dipterocarpus* leaves) achieved the highest yield (43.86 g per bag) and BE (37.59%). The fruiting bodies showed favorable nutritional properties, including high carbohydrate, moderate protein, and low-fat contents. These findings establish *A. heimuer* as a newly recorded, cultivable mushroom species in Thailand and provide fundamental data on its optimal cultivation parameters, suitable spawn substrates, and nutritional value to support its further development for local mushroom production.

## 1. Introduction

*Auricularia* is a large genus in the family Auriculariaceae (Auriculariales, Agaricomycetes, Basidiomycota), which has a global distribution across the temperate, subtropical and tropical regions [1,2,3,4,5]. Species within this genus are typically found growing on decaying branches or logs in nature, playing an important role as decomposers in terrestrial ecosystems [1,2,3,4,6]. Traditionally, the classification of *Auricularia* species relied solely on morphological characteristics [7], nevertheless, the physical characteristics of those mushrooms generally vary under different environmental conditions. Recently, the combination of molecular data with morphological observations has substantially improved the accuracy of species delimitation [3,4,6,8,9]. Currently, 37 *Auricularia* species have been reported worldwide [6]. Among these, commercially important species such as *A. cornea* and *A. heimuer* are widely cultivated in China and Southeastern Asia [6]. In Thailand, 14 species have been recorded. Several species, including *A. asiatica*, *A. cornea*, *A. fibrillifera*, *A. thailandica*, *A. sinodelicata*, and *A. villosula* have been more precisely described based on both morphology and molecular phylogeny [3,4,10,11]. However, other reported species, namely *A. auricula-judae*, *A. delicata*, *A. fuscosuccinea*, *A. mesenterica*, *A. nigricans*, *A. ornata*, *A. peltate*, and *A. tenuis*, require further taxonomic confirmation [11]. When it comes to *Auricularia heimuer*, the species described in our study, was first introduced by Wu et al. [8]. Previously, specimens collected from China and East Asia were identified as *A. auricula-judae* [12]. Phylogenetic analyses based on ITS and LSU sequences revealed that these Asian samples formed a well-resolved clade separate from European samples, thereby suggesting *A. heimuer* as a distinct species [8]. Morphologically, *A. heimuer* differs from *A. auricula-judae* in having shorter abhymenial hairs, smaller basidiospores and the presence of medulla [6,8].

*Auricularia* mushrooms have been cultivated and consumed for centuries, especially in Asian countries such as China, Japan, Thailand, and Vietnam [13,14]. In addition to their culinary value, *Auricularia* species are recognized for their nutritional composition and medicinal potential. *Auricularia* species are rich in proteins, dietary fiber, vitamins, minerals, and bioactive polysaccharides that exhibit antioxidant, immunomodulatory, antimicrobial, anticoagulant, hypolipidemic, and antitumor activities [15,16,17,18]. The cultivation of *Auricularia* spp. is therefore important not only for food production but also for the development of functional food and mushroom-based bioresources. Efficient cultivation depends on appropriate environmental factors such as temperature, pH, humidity, aeration, light intensity and substrate composition, which influence mycelial colonization, fruiting body development, yield, and production consistency [13,19,20]. Substrate optimization has focused on the utilization of agricultural wastes including corn cobs (CC), cottonseed hulls, hardwood sawdust, rice straw, rubber wood, and sawdust, supplemented with rice bran or wheat bran to enhance yield and reduce cultivation costs [21,22]. In Thailand, optimization studies mainly emphasize the use of locally available agricultural residues and suitable tropical cultivation conditions to support sustainable commercial production [10,19,20].

Among cultivated species, *A. heimuer* is particularly important because it is one of the major commercially cultivated wood ear mushrooms and the third-largest cultivated edible fungus worldwide, with recognized ecological value, economic value, and nutritional and medicinal properties, and a unique taste [23]. However, cultivation productivity varies significantly between strains because of differences in nutrient utilization, environmental tolerance, mycelial growth rate, and substrate colonization ability. Therefore, cultivation protocols developed for strains from other geographical regions may not apply directly to newly isolated strains, highlighting the need for strain-specific evaluation and optimization.

To date, *A. heimuer* has not been reported in Thailand, indicating an unknown distribution and a lack of cultivation protocols. During ongoing surveys of edible mushrooms in northern Thailand, three *Auricularia* strains were isolated and characterized. This study hypothesized that (i) mycelial growth and cultivation performance of the three *A*. *heimuer* isolates vary with culture medium composition and environmental conditions, including temperature and initial pH, (ii) different nitrogen sources and inoculum types would significantly affect mycelial growth and spawn production, and (iii) optimizing these factors would improve colonization and cultivation performance. Thus, this study aimed to determine the taxonomic identity of the isolated strains through morphological and phylogenetic analyses, providing the first confirmed record of *A*. *heimuer* in Thailand. Furthermore, we sought to optimize culture conditions and inoculum types to maximize mycelial growth and inoculum production, and subsequently evaluate the cultivation performance and nutritional profile of the harvested fruiting bodies.

## 2. Materials and Methods

### 2.1. Sample Collection and Isolation

Fresh specimens of *Auricularia* were collected from dipterocarp community forests in northern Thailand during the rainy seasons (June to August) of 2024. Basidiomata were photographed on-site, and essential field data (location, forest type, substrates, and hosts) were recorded. The specimens were then placed in zip-lock plastic bags and transported to the laboratory for further study. Fungal cultures were isolated by cutting a piece of fertile tissue from the fruiting bodies as soon as they were brought to the laboratory, cultured on potato dextrose agar (Conda, Madrid, Spain) and incubated at 30 °C for 3–5 days in the dark. The pure cultures were deposited in the Saisamorn Lumyong’s Laboratory Culture Collection (SLLC-CMU), Faculty of Science, Chiang Mai University, Chiang Mai, Thailand, and the Mae Fah Luang University Culture Collection (MFLUCC), Mae Fah Luang University, Chiang Rai, Thailand. Meanwhile, the fruiting bodies of *Auricularia* were dried in hot-air oven at 45–50 °C to prepare herbarium materials and deposited at the Saisamorn Lumyong Laboratory Herbarium Collection (SLLH-CMU), Faculty of Science, Chiang Mai University, Chiang Mai, Thailand, and the herbarium of Mae Fah Luang University (MFLU), Mae Fah Luang University, Chiang Rai, Thailand.

### 2.2. Fungal Strains

Three different *A. heimuer* strains (SL115, SL150, and SL160) were used in the study. Dried specimens obtained from the SLLH-CMU collection were used for morphological characterization, while pure cultures from the SLLC-CMU collection were used for molecular analysis, optimization of mycelial growth conditions, and evaluation of suitable inoculum types.

### 2.3. Morphological Examination

Macromorphological characteristics were observed using fresh specimens with a Nikon SMZ800N stereo microscope (Nikon Instruments Inc., Melville, NY, USA). The dried mushrooms were cut cross-sectionally and prepared as permanent slides by staining with lactophenol cotton blue (to observe the structure of basidiospores). Micromorphological characteristics were examined using a Nikon Eclipse Ni with a Nikon DS-Ri2 camera system (Nikon Instruments Inc., Melville, NY, USA). Measurements of various features were recorded using Tarosoft^®^ Image Frame Work software version 0.9.7. Photographic plates were prepared using Adobe Photoshop CS5 Extended version 12.0 software (Adobe Systems, San Jose, CA, USA) to describe the morphological characteristics of the mushrooms.

### 2.4. DNA Extraction, PCR Amplification and Sequencing

The genomic DNA was extracted from fungal mycelia using an E.Z.N.A.^®^ tissue DNA kit (Omega, Norcross, Georgia). The DNA was then amplified using the Polymerase Chain Reaction (PCR) technique. The final PCR was prepared in 25 μL, consisting of 12.5 μL of 2 × Taq PCR Master Mix (Bio-helix, New Taipei City, Taiwan), 1 μL each of a forward and a reverse primer, 1 μL of extracted DNA, and 11.5 μL of double-distilled water. The internal transcribed spacer (ITS) region was amplified with ITS4 and ITS5 primers [24], and the nuclear large subunit (nLSU) ribosomal DNA region was amplified with LR0R and LR7 primers (https://sites.duke.edu/vilgalyslab/rdna_primers_for_fungi/, accessed on 10 January 2026). The PCR conditions for both loci were as follows: an initial denaturation step of 3 min at 94 °C, followed by 35 cycles of 30 s at 94 °C, 50 s at 55 °C and 1.30 min at 72 °C, and a final denaturation step of 10 min at 72 °C. The quality of the PCR samples was checked by agarose gel electrophoresis, and DNA concentration was determined using a NanoDrop spectrophotometer (OneC, Thermo Scientific, Waltham, MA, USA). The molecular sequencing analysis was performed by 1st Base (Seri Kembangan, Selangor Malaysia). The newly obtained sequences were submitted to the NCBI database to obtain GenBank accession numbers.

### 2.5. Phylogenetic Analysis

The generated nucleotide sequences were subjected to BLAST searches to check their similarity to sequences of *Auricularia* in the GenBank database (http://www.ncbi.nlm.nih.gov/blast/, accessed on 10 January 2026) and downloaded based on previous studies [8,11] (Supplementary Table S1). Multiple sequence alignment was conducted using the MAFFT v7.307 online version [25] (https:// mafft.cbrc.jp/alignment/server/, accessed on 10 January 2026) and manually modified using BioEdit version 7.0.5.3 [26]. The combined ITS-LSU phylogenetic trees were constructed via Maximum Likelihood (ML) using RAxML-HPC2 on XSEDE (v.8.2.12) and Bayesian inference (BI) analyses using MrBayes on XSEDE v.3.2.7a via the CIPRES Science Gateway platform V3.3 [27,28,29]. The GTRGAMMA model of the bootstrapping phase with 1000 bootstrap iterations was set as the parameter for ML analysis [29]. Bayesian posterior probability (BYPP) for sequence information was used for phylogenetic analyses of *Auricularia* in the present study.

### 2.6. Determination of Mycelial Growth Under Different Culture Conditions

After each experiment was prepared, all culture media were sterilized by autoclaving at 121 °C for 15 min, whereas non-synthetic media prepared from lignocellulosic residues were autoclaved for 30 min to ensure complete sterilization. After sterilization, the culture media were poured into 90 mm diameter plastic Petri dishes and allowed to solidify, after which a sterile cellophane sheet was carefully placed onto the surface of the medium in each plate. A 5 mm diameter mycelial plug was cut from the edge of 5-day-old colonies grown on PDA incubated at 30 °C and transferred aseptically to the center of each medium plate. After 7 days of inoculation at 30 °C, the colony diameter was measured by the two-perpendicular-diameter method, and mycelial growth rate (mm day^−1^) was calculated [30]. Fungal growth was also evaluated in terms of biomass production (mg dry weight). Mycelial dry weight was measured after the cellophane disks were dried at 60 °C overnight or until a constant weight was obtained, and represented the final dry biomass [31]. Each experiment was performed with five replicates. Mycelium growth was characterized based on growth rate (mm day^−1^) and mycelial density. Mycelial density was categorized as high (+++), moderate (++), or low (+) [32].

#### 2.6.1. Effects of Different Media on Mycelium Growth

Eight different agar-based media were tested for their ability to support mycelial growth of *A. heimuer*. Three of these were synthetic media, including PDA, yeast extract agar (YEA), and mushroom complete media agar (MCMA). The other five were prepared as non-synthetic media from lignocellulosic residues, including corn cob agar (CCA), corn dust agar (CDA), corn stalk agar (CSA), *Dipterocarpus* leaves agar (DLA), and rice straw agar (RSA). For the preparation of non-synthetic media, lignocellulosic materials were first extracted in distilled water at a ratio of 1:10 (*w*/*v*) and allowed to soak at room temperature for 24 h. The mixture was then filtered, and 1000 mL of supernatant was supplemented with 10 g glucose and 2 g of agar [23,33]. All media were sterilized by autoclaving at 121 °C for 30 min, following the method described by Zurbano et al. [33]. A sterilized cellophane disk was placed on the surface of the tested culture media. The medium that presented the highest mycelial growth rate and biomass yield was selected for further experiments.

#### 2.6.2. Effect of Temperature on Mycelium Growth

To determine the optimal temperature for mycelial growth, a mycelium disc of each *A. heimuer* strain was aseptically inoculated (as explained before) onto the respective optimal medium. The inoculated plates were then incubated in darkness under controlled temperature conditions of 20, 25, 30, and 35 °C. Growth performance was assessed to determine the effect of temperature on mycelial growth and the optimal temperature range for each strain. Mycelial growth was evaluated by measuring colony diameter, while biomass accumulation was quantified as dry weight after seven days of incubation.

#### 2.6.3. Effect of Initial pH Value of Culture Medium on Mycelium Growth

In this experiment, the previously determined optimal culture medium for each *A. heimuer* strain was used to evaluate the effect of initial pH on mycelial growth. The pH of each suitable medium for each strain was adjusted to 4.0, 5.0, 6.0, 7.0, 8.0, and 9.0 using 0.1 M sodium hydroxide (NaOH) or 0.1 M hydrochloric acid (HCl) before sterilization by autoclaving. Following sterilization, each medium was aseptically inoculated with the respective fungal strain. The inoculated cultures were then incubated in the dark at the previously determined optimal temperature specific to each strain. Mycelial growth rate was determined by measuring colony diameter, while biomass production was assessed as dry weight after seven days of incubation.

#### 2.6.4. Effect of Nitrogen Sources on Mycelium Growth

Here, CDA was used as a basal medium to evaluate optimal nitrogen sources for mycelial growth. Nine nitrogen sources were tested, including potassium nitrate (KNO_3_), ammonium chloride (NH_4_Cl), ammonium sulfate [(NH_4_)_2_SO_4_], sodium nitrate (NaNO_3_), malt extract, peptone, tryptone, yeast extract, and urea. Each nitrogen source was supplemented individually at 1% (*w*/*v*). Before sterilization, the pH of each medium was adjusted to the previously determined optimal value. All media were autoclaved, poured and inoculated under aseptic conditions. Mycelial growth rate and dry biomass were determined after seven days of incubation in the dark at the optimal temperature for mycelial growth.

### 2.7. Determination of Mycelial Growth on Different Types of Mushroom Spawn

#### 2.7.1. Sources of Material and Preparation

In this experiment, lignocellulosic biomass derived from agricultural and natural residues was used. Material used for solid spawn included CC, *Dipterocarpus* leaves (DL), rubber sawdust (RS), and spent coffee grounds (SCG). For stick spawn, the substrates comprised banana leaf sheath (BLS), corn stalk (CS), lotus stalk (LS), water hyacinth stalk (WHS), longan stalk (LGS), giant mimosa stalk (GMS), and rubber tree wood (RTW). All materials were collected from commercial agricultural fields and forested areas in Chiang Mai, northern Thailand.

Prior to use as substrates for inoculum preparation, lignocellulosic materials designated for solid spawn were initially air-dried to reduce moisture content, then were subsequently reduced in size with a wood chipper and sieved to obtain particles ranging from 5 to 10 mm in diameter [34]. Materials intended for stick spawn were manually cut to approximately 10 cm in length and subsequently dried in a hot-air oven at 60 °C until a stable weight was achieved, following the method described by Xayavong et al. [35].

#### 2.7.2. Preparation of Spawn and Inoculation

The mushroom spawn was evaluated using four different inoculum types, including grain, solid, liquid, and stick, to determine the effects on mycelial colonization. Each type of spawn was prepared using different base materials, as described below.

For grain spawn preparation, various grains, including red bean (RB), mung bean (MB), corn kernel (CK), sorghum seed (SS), paddy rice (PR), and barley (BL) were thoroughly washed with running tap water and boiled for 10–15 min or until grains became soft. Excess water was drained, and the grains were then air-dried. Solid spawn substrates (CC, DL, RS, and SCG) were adjusted to a moisture content of approximately 60–70%. Both grain and solid substrates were packed into glass test tubes (18 mm in diameter × 180 mm long) to a height of 10 cm. For liquid spawn, the liquid culture medium was prepared following the same procedures previously used for evaluating the effects of different media on mycelial growth, including both synthetic (PD, YE, and MCM) and non-synthetic (CC, CD, CS, DL, and RS). After extraction and filtration, 5 mL of the supernatant from each medium was transferred into glass test tubes (18 mm in diameter × 180 mm long). Each tube was inoculated with the tested fungal strain and incubated in a reciprocal shaker at room temperature (approximately 25–28 °C) at 150 rpm. Stick spawn was prepared by immersing each material overnight in water, to achieve a moisture content of approximately 60–65% [35]. After that the materials were placed into 18 mm in diameter × 180 mm long glass test tubes.

After preparation of each spawn type, all prepared substrates were sterilized by autoclaving at 121 °C for 30 min. After cooling to room temperature, each substrate was inoculated with mycelial plugs (5 mm in diameter), obtained from actively growing cultures of each *A. heimuer* strain for grain and solid spawn, while mycelial plugs (10 mm in diameter) were used for stick spawn and incubated at 30 °C in the dark. For liquid spawn preparation, five mycelial plugs (5 mm) were inoculated into each tube. All inoculated cultures were incubated on a shaker at 150 rpm for 7 days. Subsequently, the mycelial biomass was harvested, and dry weight was determined after drying at 60 °C for 24 h or until a constant weight was achieved [36].

#### 2.7.3. Determination of Mycelial Growth

Mycelial colonization was determined by daily measuring of linear mycelial extension within culture tubes using a digital caliper over a 14-day incubation period. The growth rate (mm day^−1^) was estimated from the linear part of the plot of the length against incubation time [37]. The average growth rate values of 5 replicates were reported for each treatment. After 14 days of incubation, mycelial density was classified as thick (+++), thin (++), or very thin (+), following the methodology described by Schoder et al. [38].

### 2.8. Preliminary Cultivation on Different Substrates for Fruiting Body Induction

Based on the optimal culture conditions, two *A. heimuer* strains exhibiting comparatively superior mycelial growth and biomass production were selected for subsequent experiments on the effects of different inoculum types and preliminary cultivation trials. The study aimed to compare quantitative and qualitative production characteristics of these strains with those of the standard strain (*A. cornea* TMCC No. 8) obtained from the Department of Agriculture, Bangkok, Thailand, and a commercially cultivated strain (*A. cornea* SL167) obtained from the SLLC-CMU collection. The selected strains exhibited superior mycelial growth rates and mycelial density during previous screening experiments, indicating strong potential for commercial cultivation.

#### 2.8.1. Substrate Preparation and Spawn Production

Rubber tree sawdust was used as the primary substrate and served as the control treatment. The substrate was supplemented with 5% rice bran, 1% calcium carbonate, 2% calcium sulfate, and 0.2% sodium sulfate (*w*/*w*), following a modified formulation adapted from Walker et al. [19]. To evaluate the potential of agricultural and forestry residues as alternative substrates, corn stalks, *Dipterocarpus* leaves, sugarcane leaves, and teak leaves were each mixed with rubber tree sawdust at a 50:50 ratio (*w*/*w*) and supplemented with the same nutrient formulation.

All substrate mixtures were thoroughly blended and adjusted to a moisture content of approximately 60–70%. The prepared substrates (600 per bag) were packed into polypropylene cultivation bags fitted with plastic rings and lids. The bags were sterilized at 121 °C for one hour and allowed to cool to room temperature (approximately 25 °C). Subsequently, 5 g of SS spawn inoculum was aseptically inoculated into each bag under sterile conditions. The inoculated bags were incubated in darkness at 25 ± 3 °C until complete mycelial colonization was achieved. The time required for full colonization and contamination incidence was recorded for each treatment. Twenty replicate bags were prepared for each treatment.

#### 2.8.2. Analysis of Nutritional Composition

Dried fruiting body samples of the high-potential strain *A. heimuer*, cultivated on various substrate mixtures, were subjected to proximate composition analysis and compared with those of *A. cornea*. The nutritional evaluation focused on the major chemical constituents, including moisture content, ash, crude fat, crude fiber, crude protein, total carbohydrates, and energy value. Analyses were performed according to standard procedures established by the Association of Official Analytical Chemists (AOAC), including crude protein (AOAC 991.20), crude fat (AOAC 948.15), ash (AOAC 923.03 and 920.153), moisture content (AOAC 925.10 and 950.46), and crude fiber (internal method TE-CH-122 based on AOAC 978.10). Total carbohydrate content was determined following the nutritional labeling analytical approach expressed by Walker et al. [19]. The energy value of each sample was estimated using bomb calorimetry following AOAC-based procedures for food energy determination. All chemical analyses were conducted at the Central Laboratory and the Animal Nutrition Laboratory, Department of Animal and Aquatic Sciences, Faculty of Agriculture, Chiang Mai University, Thailand.

### 2.9. Statistical Analysis

All data were analyzed using one-way analysis of variance (ANOVA) with IBM SPSS Statistics software (version 26.0). The results were expressed as means ± standard deviation (SD). Differences between the mean value of each treatment, measured using Duncan’s multiple-range test, were considered statistically significant if *p* ≤ 0.05.

## 3. Results and Discussion

### 3.1. Molecular Identification and Phylogenetic Placement

**Figure 1:**
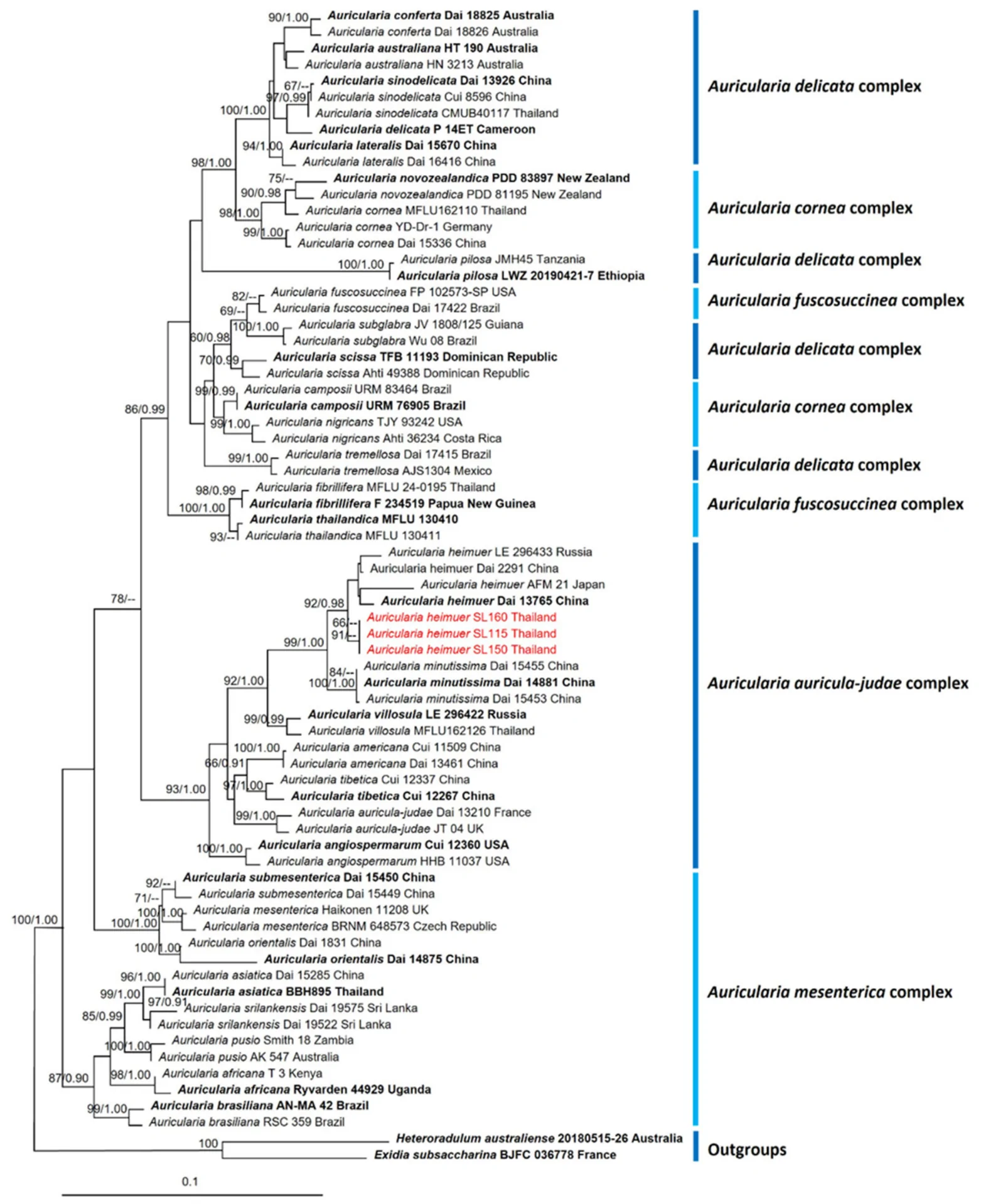
Phylogenetic tree inferred from RAxML analyses of combined ITS and nLSU sequence alignments. ML bootstrap values ≥ 60% (left) and Bayesian inference posterior probability ≥ 90% (right) are given at the nodes. The tree is rooted to *Exidia subsaccharina* BJFC 036778 and *Heteroradulum australiense* 20180515-26. Ex-type strains are in bold. New records are indicated in red.

Phylogenetic analysis of combined ITS and nLSU sequence data comprised 71 strains of *Auricularia*, including *Exidia subsaccharina* BJFC 036778 and *Heteroradulum australiense* 20180515-26 as the outgroups (Figure 1). The RAxML analysis obtained the best-scoring tree with a final ML optimization likelihood value of −8318.652496. The final matrix contained 406 distinct alignment patterns, with 17.88% undetermined characters or gaps. The estimated base frequencies were recorded as follows: A = 0.249114, C = 0.229770, G = 0.278012, T = 0.243104; substitution rates AC= 1.895135, AG = 1.945162, AT = 2.064810, CG = 0.983863, CT = 7.169766, and GT = 1.000000. The average SD of split frequencies for Bayesian analysis was 0.009729. The topologies generated by ML and BI analyses were consistent; thus, the RAxML tree is used as a backbone tree. Based on the ML analysis of the combined ITS and LSU sequences, *Auricularia* species are divided into four major clades, corresponding to the *A. auricula-judae*, *A. cornea*, *A. delicata*, *A. fuscosuccinea* and *A. mesenterica* species complexes (Figure 1), similarly to previous studies [6,11]. The newly obtained strains (SL115, SL150, SL160) were placed within the *A. auricula-judae* complex and formed a well-supported sister clade (92% ML and 0.98 BYPP support) to *A. minutissima* with 99% ML and 1.00 BYPP support (Figure 1).

**Figure 2:**
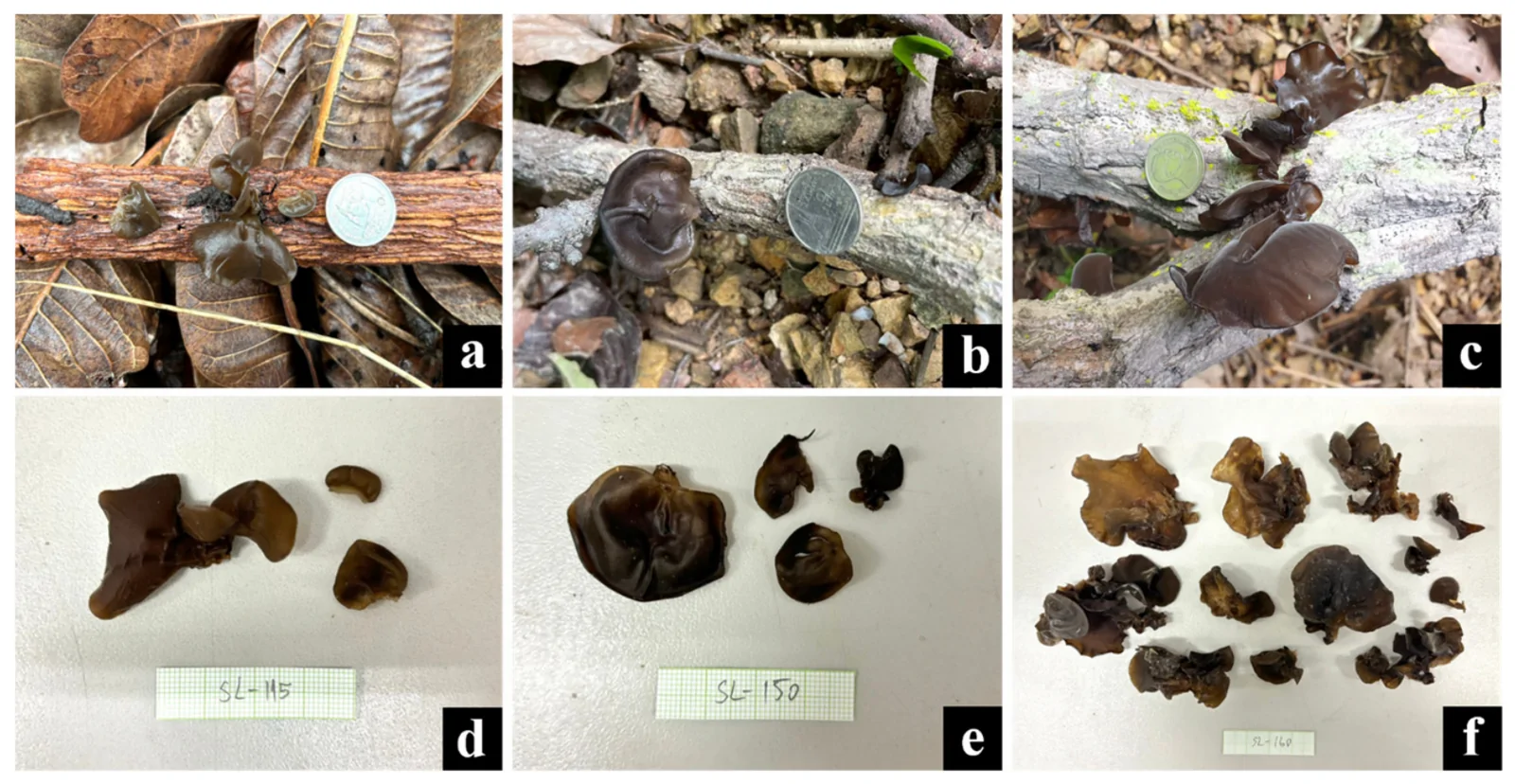
Macroscopic structures of *Auricularia heimuer*. (**a**,**d**) *A. heimuer* strain SL115; (**b**,**e**) *A. heimuer* strain SL150; (**c**,**f**) *A. heimuer* strain SL160.

**Figure 3:**
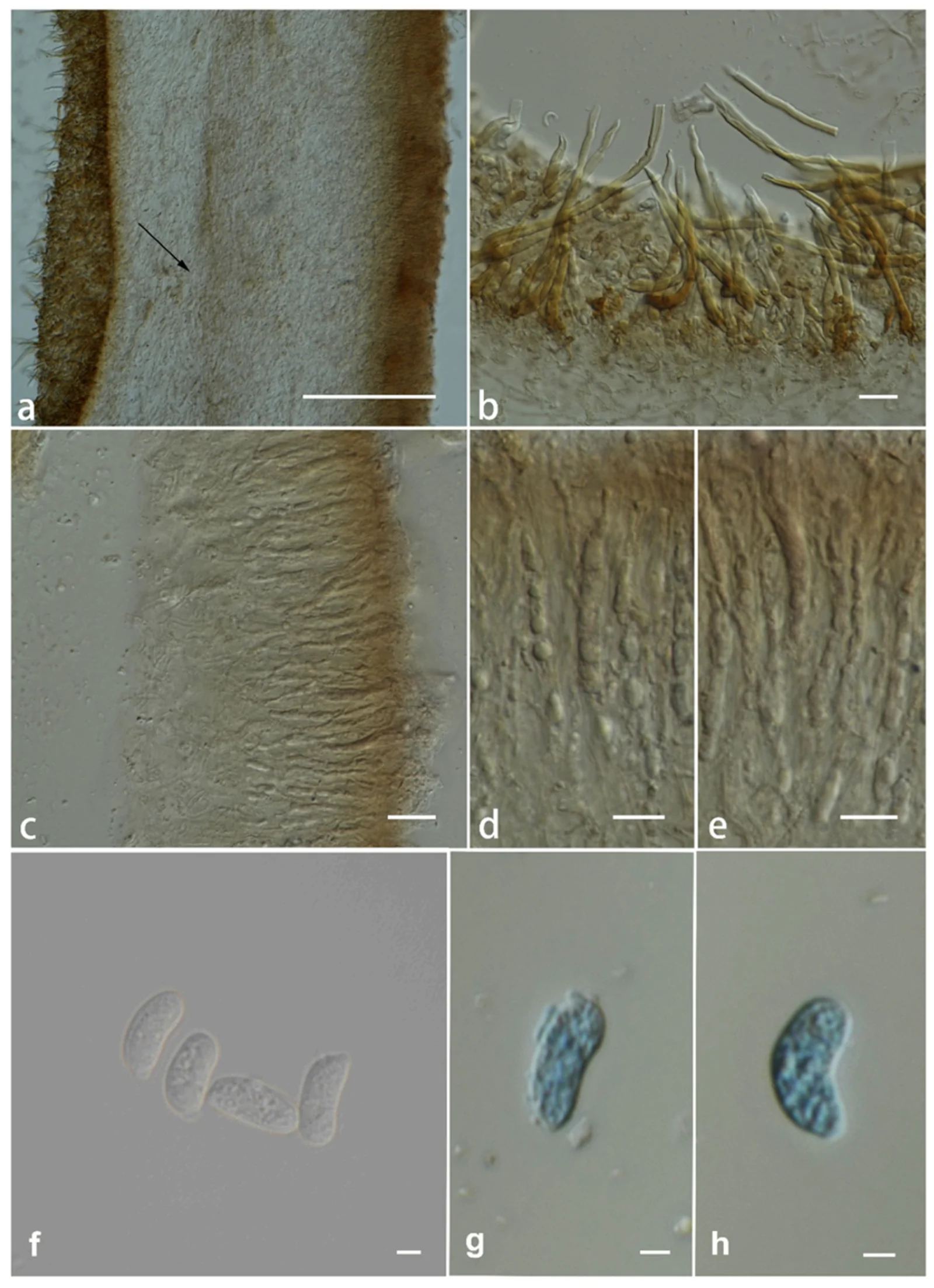
Microscopic structures of *Auricularia heimuer* (SL160). (**a**) Cross-section of a basidioma (an arrow shows the medulla); (**b**) abhymenial hairs; (**c**–**e**) basidia and basidioles; (**f**–**h**) basidiospores. Bars: (**a**) 300 µm; (**b**,**c**) 20 µm; (**d**,**e)** 10 µm; (**f**–**h**) 2 µm.

**Taxonomy**
*Auricularia heimuer* F. Wu, B.K. Cui and Y.C. Dai, Phytotaxa 186: 248 (2014)
Index Fungorum number: IF809995, Figure 2 and Figure 3.
**Basidiomata**—Gelatinous when fresh, solitary or caespitose, sessible or substipitate, auriculiform to petaloid with undulate margin, when mature up to 13 cm broad, 0.8–1.5 mm thick; pilose with folds, light brown (6D4) to reddish brown (8E5) when fresh, grayish brown (6D3–8F5) when dry; hymenial surface to venose with few folds, claybuff when fresh and grayish violet (6C5) to blackish gray (G1), coal black (5H2) when dry.
**Internal features**—Medulla present in the middle of the cross-section; abhymenial hairs, 50–150 × 3–6.5 µm, irregular, hyaline, thick-walled with a narrow lumen, apically acute or obtuse, usually single, sometimes tufted; basidia clavate, transversely 3-septate, with oil guttules, sterigmata rarely observed, 36–67 × 3.0–6.5 µm. Basidiospores allantoid, hyaline, thin-walled, smooth, usually bearing one or two large guttules, IKI-, CB-, (11.4–)11.7–12.8(–14.0) × (4.5–)4.6–5.4(–5.9) µm, L = 12.2 µm, W = 5.0 µm, Q = 2.27–2.64 (n = 30/1).
**Material examined**—THAILAND, Phayao Province, Bor Sib Song, Tambon Ban Tom, Amphoe Muang, 19°14′03.9″ N 99°54′06.6″ E, on decaying wood in a dipterocarp forest, 15 July 2024; W. Aiduang, K. Jatuwong, J. Monkai & T. Thitla, SL115 (MFLU 26-0326), living culture, MFLUCC 26-0298, Lampang province, Wat Phra That Doi Phra Chan, Tambon Pa Tun, Amphoe Mae Tha, on decaying wood in a dipterocarp forest, 26 August 2024; W. Aiduang, K. Jatuwong, J. Monkai, W. Phonrob & T. Thitla, SL150 (MFLU 26-0327), living culture, MFLUCC 26-0299, Lampang Province, Wat Phra That Doi Phra Chan, Tambon Pa Tun, Amphoe Mae Tha, on decaying wood in a dipterocarp forest, 26 August 2024; W. Aiduang, K. Jatuwong, J. Monkai, W. Phonrob & T. Thitla, SL160 (MFLU 26-0328), living culture, MFLUCC 26-0300.
**Distribution**—China, Japan, Russia, Thailand [6,8, this study].
**Notes**—According to our phylogenetic analysis, the new strains (SL115, SL150, SL160) clustered in a single lineage with *A. heimuer* with strong support (92% ML and 0.98 BYPP) and this lineage is sister to *A. minutissima* (99% ML and 1.00 BYPP support; Figure 1). Morphologically, our new collections are consistent with the original description of *A. heimuer* from China [8]. The basidiomata are gelatinous and reddish-brown when fresh, with an upper surface pilose with folds, and a well-developed medulla. In addition, the size of abhymenium hairs (50–150 × 3–6.5 vs. 50–150 × 4–6.5 µm) and basidia (36–67 × 3.0–6.5 µm vs. 40–67 × 3.0–6.5 µm) overlap with those reported for the type specimen [8], whereas basidiospores of our samples were slightly larger than those of the type specimen (11–14 × 4.5–6 vs. 11–13 × 4–5 µm) [8]. Although *A. heimuer* is phylogenetically closely related to *A. minutissima*, the latter can be distinguished by the presence of two types of basidiomata, smaller basidiomata (up to 12 cm vs. up to 4) and larger basidiospores (11–13 × 4–5 vs. 12–16 × 5–7 µm) [6,9]. A morphological comparison between Thai collections and type specimens of *A. heimuer* and *A. minutissima* is provided in Supplementary Table S2.

This study presents the first record of *A. heimuer* in Thailand, supported by detailed morphological characteristics and phylogenetic analyses. Recent multilocus phylogenetic studies based on combined ITS, LSU, RPB1, and RPB2 datasets have demonstrated that multiple loci are useful for resolving the taxonomic relationships among closely related *Auricularia* species [6]. In the present study, we were unable to successfully amplify the protein-coding genes RPB1 and RPB2 from the newly collected Thai specimens. Nevertheless, the combined ITS + nLSU phylogeny, together with the congruent morphological characteristics of the specimens, supports their identification as *A. heimuer*. However, given that A. heimuer belongs to the A. auricula-judae species complex, relying on only two loci constrains phylogenetic resolution and represents a limitation of the current identification. Thus, future studies incorporating expanded multilocus datasets, particularly RPB1 and RPB2, will be necessary to further confirm the taxonomic placement of the Thai collections, improve species-level phylogenetic resolution, and evaluate intraspecific diversity within *A. heimuer*.

The morphology of our wild Thai collections closely matches that of wild *A. heimuer* strains, including the holotype described from China [8]. Compared to temperate Chinese cultivated strains, basidiomata of wild *A. heimuer* are generally lighter in color when fresh and dried, and become thinner upon drying. In addition, the abhymenial hairs in wild specimens tend to be shorter and more sparsely distributed [8]. In this study, cultivated *A. heimuer* also exhibited darker basidiomata, consistent with previous observations; however, they were thinner than the wild specimens. These differences further support the impact of environmental factors on morphological characteristics in *Auricularia* species.

Ecologically, *Auricularia heimuer* has been reported to occur in boreal and temperate regions of Northern China, Japan and Russia, where it is commonly associated with decaying wood of Quercus (Fagaceae) [8]. Our finding significantly expands its known geographical distribution into tropical regions and reveals a new host association with Dipterocarpaceae. These results highlight the ecological adaptability of *A. heimuer* across diverse climatic zones and host substrates.

On the one hand, taxonomic studies of *Auricularia* in Thailand have increased since 2017, leading to reports of new species such as *A. asiatica* and *A. thailandica*, as well as new records, including *A. cornea*, *A. fibrillifera*, *A. sinodelicata* and *A. villosula* [3,4,10,11]. On the other hand, further investigation of commercially cultivated strains currently identified as *A. auricula-judae* in Thailand and Southeast Asia is necessary to clarify their taxonomic status.

### 3.2. Mycelial Growth Tests of A. heimuer

#### 3.2.1. Effects of Different Culture Media

**Figure 4:**
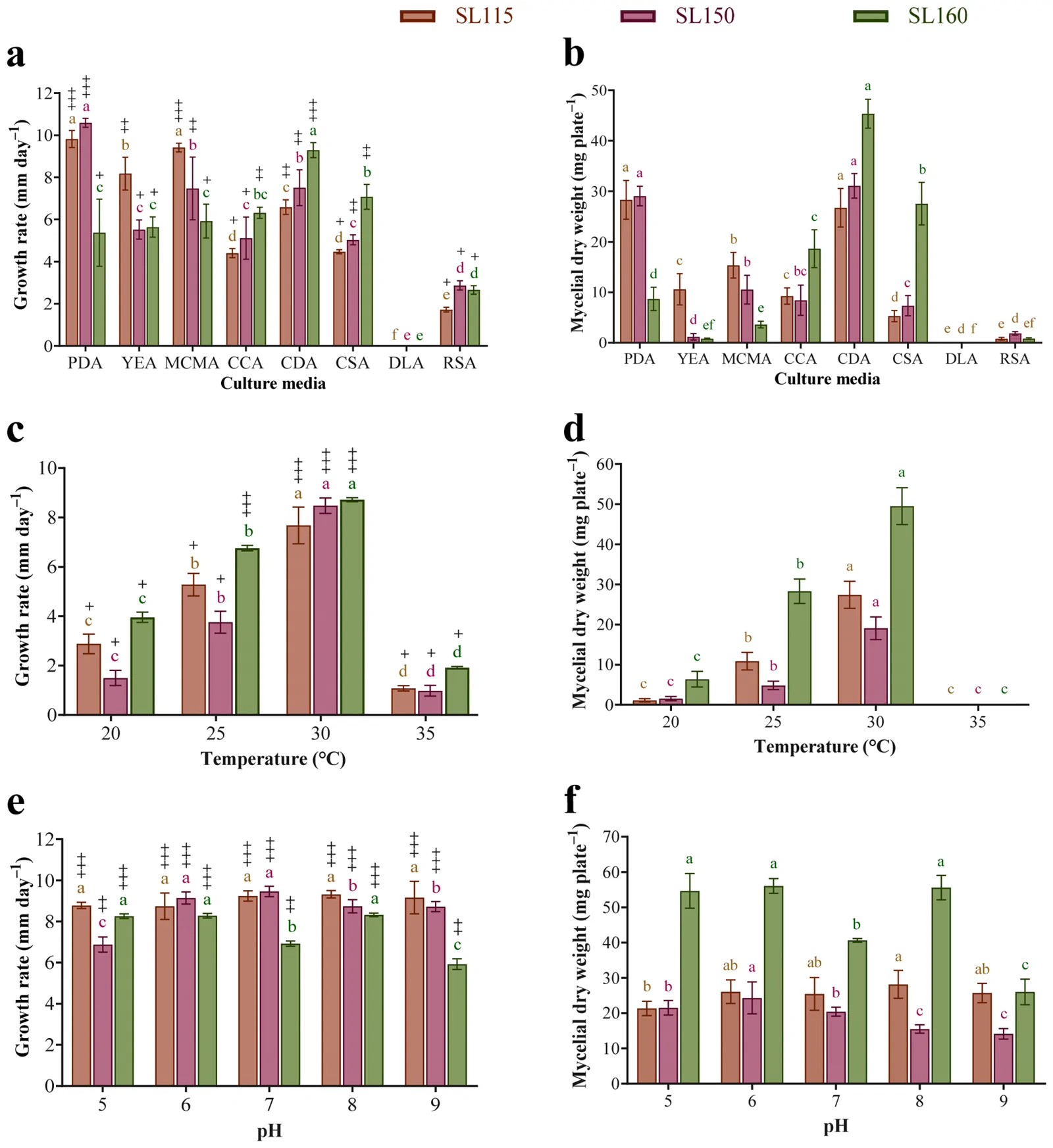
Comparison of mycelial growth and dried weight of *Auricularia heimuer* after 14 days under different conditions: (**a**,**b**) different culture media; (**c**,**d**) at different pH levels; and (**e**,**f**) different incubation temperatures. Note: CCA = corncob agar; CDA = corn dust agar; CSA = corn stalk agar; DLA = *Dipterocarpus* leaf agar; RSA = rice straw agar; PDA = potato dextrose agar; YEA = yeast extract agar; MCMA = mushroom complete media agar. The results are presented as means ± SD (n = 5). Data with different letters within the same experiment group indicate significant difference at *p* ≤ 0.05 according to Duncan’s multiple-range test. The symbol “+” represents the density of mycelial growth on the medium, where +++ = high density, ++ = moderate density, and + = low density.

The mycelial growth of three *A. heimuer* strains from the SLLC (SL115, SL150, and SL160) was evaluated on eight different culture media to determine the optimal medium for mycelial growth. The results showed that both mycelial growth rate and dry biomass production varied significantly among the three strains depending on the culture medium used, indicating that nutrient composition plays a critical role in fungal growth. The effects of different culture media on mycelial growth are presented in Figure 4a,b. After seven days of incubation, SL115 exhibited the highest growth rate on PDA at 9.82 ± 0.40 mm day^−1^, which was not significantly different from that observed on MCMA (9.42 ± 0.20 mm day^−1^). However, the highest mycelial dry weight for this strain was obtained on PDA (28.3 ± 3.83 mg plate^−1^), compared with 15.4 ± 2.54 mg plate^−1^ on MCMA. Similarly, SL150 showed the best performance on PDA, with a growth rate of 9.42 ± 0.20 mm day^−1^ and a dry weight of 29.1 ± 1.93 mg plate^−1^. In contrast, SL160 showed the highest growth on CDA, reaching a growth rate of 10.58 ± 0.21 mm day^−1^ and the highest biomass production of 45.4 ± 2.86 mg plate^−1^. Notably, none of the strains were able to grow on DLA, suggesting that this medium is unsuitable for the mycelial growth of *A. heimuer*.

Previous studies have reported that mycelial growth of edible mushrooms is significantly influenced by culture media composition, as differences in nutrient availability can influence growth rate, mycelial density, and biomass accumulation [19,32,39,40]. Several studies have also revealed that PDA is a suitable medium for the cultivation of various mushroom species, including *Pleurotus* species [39,41], *Morchella rufobrunnea* [42] and *Trametes versicolor* [43]. However, species within the genus *Auricularia* show different responses depending on the culture medium used. For example, Zurbano et al. [33] reported that *A. polytricha* cultured on coconut water gelatin exhibited the fastest mycelial ramification, highest growth rate, and densest mycelial development. Similarly, Walker et al. [19] mentioned that *A. cornea* grows well on rice bran sucrose agar. In this study, PDA supported higher mycelial growth and biomass production in SL115 and SL150, whereas SL160 showed more effective growth on CDA. These differences in response may be associated with strain-specific differences in nutrient utilization and metabolic capacity. As a wood-decaying basidiomycete, *A. heimuer* relies on extracellular enzymes to degrade complex substrates into absorbable nutrients [44,45]. Moreover, the nutritional requirements of *A. heimuer* are not uniform among strains, even within the same species. The readily available nutrients in PDA may have favored SL115 and SL150, while corn-derived nutrients in CDA may have been more effectively utilized by SL160.

The effects of temperature on mycelial growth rate and dry weight of *A. heimuer* strains are presented in Figure 4c,d. Significant differences were observed among temperature treatments (*p* ≤ 0.05), indicating that temperature significantly influenced fungal growth and biomass production. All tested strains were able to grow within the temperature range of 20–35 °C; however, growth response varied depending on the incubation temperature. The highest mycelial growth rate and dry weight were recorded at 30 °C, followed by 25 °C, while significantly lower growth was observed at 20 °C and 35 °C. After seven days of incubation at 35 °C, all strains showed a significant decrease in both mycelial growth rate and biomass production, demonstrating that increased temperature negatively affected the growth of the three *A. heimuer* strains. Mycelial density was also high at 25–30 °C, while low mycelial density was observed at 20 °C and 35 °C. Collectively, these results indicate that 30 °C is the most suitable temperature for mycelial cultivation of the three *A. heimuer* strains examined in this study.

Temperature is considered one of the most important environmental factors affecting fungal growth, as it significantly influences fungal enzymatic activity and hyphal extension rates [46]. The current findings are consistent with several previous reports on edible and medicinal mushrooms, in which mycelial growth across various fungal species has been shown to be highly dependent on suitable temperature conditions. It has been shown that the optimum temperature within the genus *Auricularia* varies and is species-dependent. The optimal temperature for *A. auricula-judae* mycelial growth has been reported to be 25–30 °C [47], although Zervakis et al. [48] revealed that optimal growth of this species occurred at either 20 °C or 25 °C, depending on the nutrient medium used. Similarly, the optimal temperature for *A. polytricha* was found to be 25 °C [49], while Priya and Geetha [50] reported that 20 °C was optimal for both *A. polytricha* and *A. auricula*. In the case of *A. villosula*, Zhang et al. [51] reported that mycelial growth was most effective at 30 °C. Comparable findings were reported for *A. cornea*, where Sun et al. [52] found that incubation at 30 °C resulted in the fastest growth rate and the development of dense, strong mycelia. In contrast, other studies have suggested lower optimal temperatures for *A. cornea*, with Zervakis et al. [48], Walker et al. [19], and Jahedi et al. [53] reporting optimal growth at 25 °C and 28 °C, respectively. For *A. heimuer*, Nie et al. [54] recently reported that mycelial growth rate peaked at 35 °C. Nevertheless, this result differs from the findings of the present study, in which growth was significantly limited at 35 °C, possibly reflecting differences in strain origin, physiological adaptation, or culture conditions. Recent genomic evidence revealed significant geographical clustering among wild *A. heimuer* germplasms, suggesting genetic differentiation among geographical populations [55]. Accordingly, the temperature response of *A. heimuer* may vary considerably among geographical populations, potentially contributing to differences in optimal growth temperatures between Thai and Chinese strains.

The lower growth at 35 °C may be due to heat-induced physiological stress, which can affect metabolic activity and cellular stability [45,56]. In contrast, the optimal growth observed at 30 °C suggests that this tempertature provides a suitable balance of conditions, resulting in less stress on the mycelium [54].

Beyond *Auricularia*, comparable optimal temperatures have been reported for other edible and medicinal mushroom species. Studies on *Ganoderma sinense* demonstrated that the highest mycelial growth rate was observed at 25–30 °C, with no mycelial growth occurring at 35 °C [32]. Similarly, the optimal temperature for mycelial growth of *Pleurotus florida* and *Laetiporus sulphureus* was reported to be 25–30 °C [57,58]. Optimal temperatures for mycelial growth have further been reported to be 22–24 °C for *Agaricus bisporus* [59], 30–32 °C for *Calocybe indica* [60], 30 °C for *Ganoderma lucidum* [61], and 25 °C and 30 °C for *Hericium erinaceus* and *H. coralloides*, respectively [40]. Basidiomycetes can generally be categorized into low-, intermediate-, and high-temperature groups according to their optimal mycelial growth temperatures. Species in the low-temperature group typically grow best at 25–30 °C [61]. Based on the present results, *A. heimuer* exhibits characteristics similar to the low-temperature group, with optimal growth at 30 °C and significantly minimizing the growth at 35 °C. Overall, these findings highlight the importance of optimizing temperature to enhance mycelial colonization efficiency in *A. heimuer* cultivation.

#### 3.2.2. Effect of Initial pH Value of Culture Medium

Notably, pH is generally recognized as a key environmental factor influencing fungal metabolism, as it affects cell membrane function, nutrient absorption, enzyme activity, and secondary metabolite biosynthesis [62,63]. Figure 4e,f show the effect of initial pH on mycelial growth and dry weight across the three examined *A. heimuer* strains. The statistical analysis indicated that the mycelial growth rate and mycelial dry weight was significantly different among pH treatments (*p* ≤ 0.05), indicating that hydrogen ion concentration affects growth performance. All three strains were able to grow under acidic to neutral conditions, as shown by both mycelial growth rate and mycelial dry weight. For strain SL115, mycelial growth showed no significant differences in growth rate across the tested pH levels, ranging from 8.74 ± 0.64 to 9.32 ± 0.18 mm day^−1^. However, the highest mycelial dry weight was recorded at pH 8 (28.14 ± 3.98 mg plate^−1^). In contrast, SL150 exhibited optimal growth under slightly acidic to neutral conditions (pH 6–7), with the highest mycelial dry weight observed at pH 6 (24.30 ± 4.53 mg plate^−1^). In contrast, SL160 showed optimal growth at pH 5, 6, and 8, with the highest mycelial growth rate and dry weight. Collectively, these results suggest that pH is a determinant of mycelial development, with optimal conditions exhibiting notable strain-specific variation in *A*. *heimuer*.

Nonetheless, these findings align with previous studies showing that *Auricularia* species exhibit optimal mycelial growth across a relatively broad pH range. For instance, the mycelial growth and density of *A. auricula-judae* have been reported to be almost identical in the pH range of 6–9 [47]. Walker et al. [19] reported that mycelial growth of *A. cornea* (MFLUCC18-0346, MFLUCC18-0347 and MFLUCC23-0084) shows optimal growth at pH 5–7 or pH 5–6, depending on the culture medium. Another study on a wild *A. cornea* strain from China identified pH 7.0 as the most suitable condition for mycelial growth [52]. For *A. villosula*, Zhang et al. [51] reported that the highest biomass production was obtained at pH 8. In contrast, *A. polytricha* showed optimal growth at pH 5.0 [49], further highlighting that pH optima in *Auricularia* are influenced by the determining species and growth conditions. Collectively, these studies suggest that although *Auricularia* species typically prefer mildly acidic to neutral environments, their physiological responses to pH are variable and strain-dependent.

#### 3.2.3. Effect of Nitrogen Source

**Figure 5:**
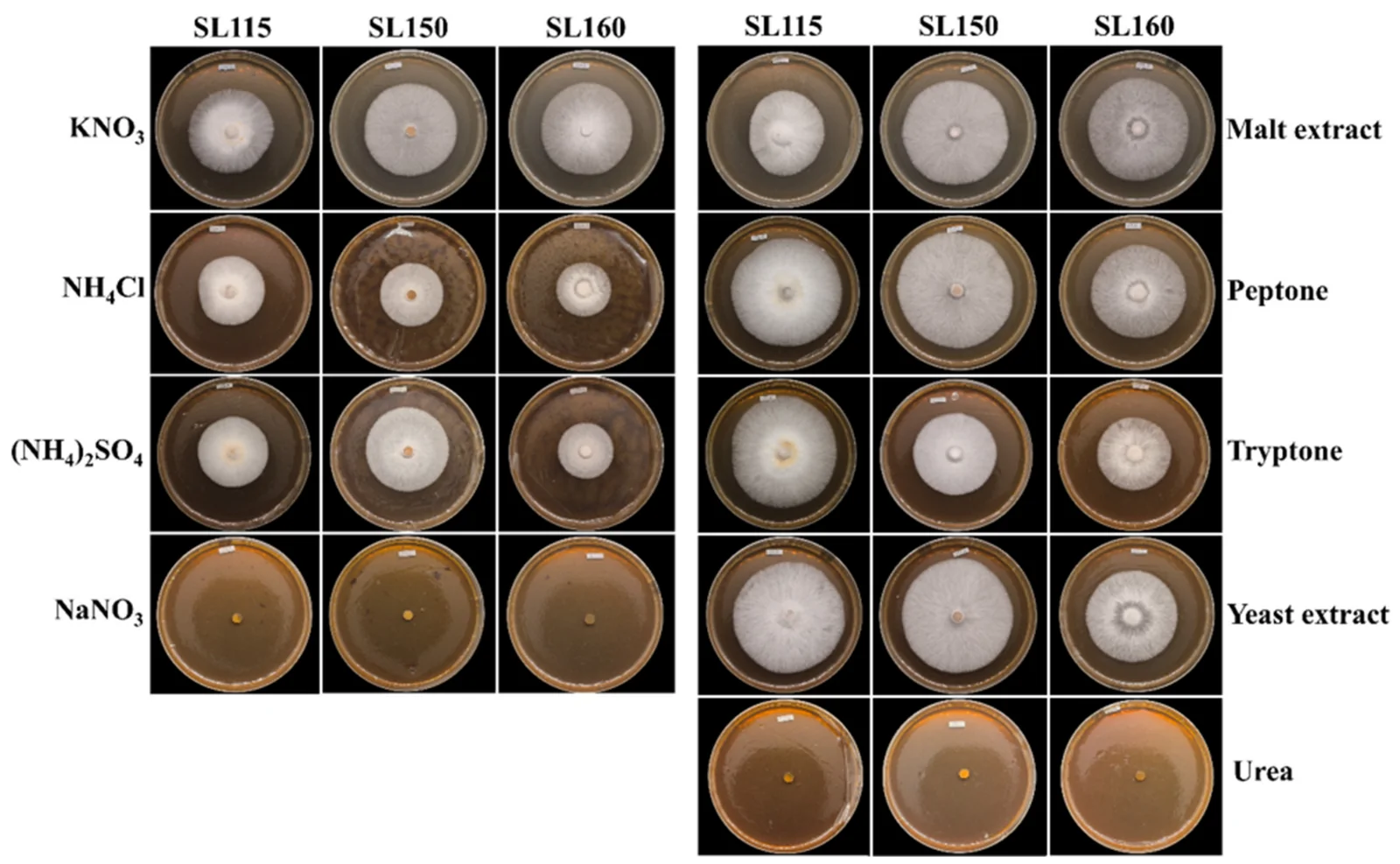
Colony morphology of *A. heimuer* cultivated on culture media supplemented with different nitrogen sources after seven days of incubation at 30 °C.

Nitrogen is an essential nutrient for fungal growth because it is required for the synthesis of many macromolecules, including proteins, nucleic acids, and chitin in the fungal cell wall [64]. Fungi are able to utilize both inorganic and organic nitrogen sources. However, the suitability of nitrogen sources varies among fungal species and strains [65,66]. In the current study, the effects of different nitrogen sources on the mycelial growth rate and biomass production of three strains of *Auricularia* spp. were evaluated using four inorganic nitrogen sources [KNO_3_, NH_4_Cl, (NH_4_)_2_SO_4_, and NaNO_3_] and five organic nitrogen sources (malt extract, peptone, tryptone, yeast extract, and urea). The results demonstrated that all tested strains responded differently to nitrogen sources (Figure 5). Most organic nitrogen sources resulted in enhanced mycelial growth and biomass production compared with inorganic nitrogen sources. Among the tested nitrogen sources, peptone, tryptone, and yeast extract yielded the highest growth rates and biomass (Table 1). For strain SL115, the highest mycelial growth rate was observed on medium supplemented with tryptone (8.62 ± 0.59 mm day^−1^), followed by yeast extract (8.18 ± 0.48 mm day^−1^) and peptone (7.82 ± 0.87 mm day^−1^). Similarly, the highest biomass dry weight was obtained from tryptone (29.38 ± 3.11 mg plate^−1^) and peptone (28.66 ± 0.81 mg plate^−1^). In strain SL150, peptone and yeast extract produced the highest growth rates (8.88 ± 0.25 and 8.48 ± 0.30 mm plate^−1^, respectively), whereas yeast extract produced the highest biomass (29.05 ± 4.54 mg plate^−1^). For strain SL160, malt extract showed the highest growth rate (8.02 ± 0.23 mm day^−1^), followed by peptone (7.28 ± 0.29 mm day^−1^) and yeast extract (7.08 ± 0.15 mm day^−1^), while peptone produced the highest biomass dry weight (24.96 ± 3.59 mm plate^−1^). In contrast, inorganic nitrogen sources resulted in lower growth rates and biomass production. KNO_3_ moderately supported mycelial growth in all strains, with growth rates ranging from 6.40 to 6.96 mm day^−1^, whereas NH_4_Cl and (NH_4_)_2_SO_4_ produced relatively lower growth rates. Notably, NaNO_3_ and urea completely inhibited mycelial growth and biomass production in all tested strains, indicating that these nitrogen sources were unsuitable for *Auricularia* mycelial development under the tested conditions.

Previous studies on *Auricularia* species have consistently reported that organic nitrogen sources are more favorable for mycelial growth than inorganic nitrogen sources. This may be attributed to the rich nutritional composition of organic nitrogen sources, particularly yeast extract, such as yeast extract which is rich in protein, amino acids, and vitamins that can support fungal cell growth [55]. For example, Jo et al. [47] reported that malt extract was the optimal nitrogen source for mycelial growth of *A. auricula-judae*. Similarly, Sun et al. [50] revealed that yeast extract was the most effective nitrogen source for promoting mycelial growth in *A. cornea*. Xu and Yun [49] also identified yeast extract as the most suitable nitrogen source for submerged culture of *A. polytricha*. Furthermore, Hassan and Medany [67] found that 0.6% yeast extract was the most favorable nitrogen source and that supplementation with this extract enhanced the mycelial biomass yield by 8.26 g/L for *A. polytricha*.

After determining the optimal environmental conditions for each strain, SL115 and SL150 were cultured on PDA at pH 7, whereas SL160 was cultured on CDA at pH 5. Each strain was supplemented with different nitrogen sources and incubated at 30 °C. The results showed that SL115 and SL150 exhibited higher mycelial growth rates and biomass production, indicating stronger potential for commercial cultivation than SL160 under their respective optimal conditions. Therefore, based on their comparatively superior mycelial growth and biomass production, SL115 and SL150 were selected for subsequent experiments on the effects of different mushroom inoculum types and cultivation performance.

### 3.3. Effect of Mycelial Growth on Different Types of Mushroom Inoculum

#### 3.3.1. Liquid Spawn Inoculum

**Figure 6:**
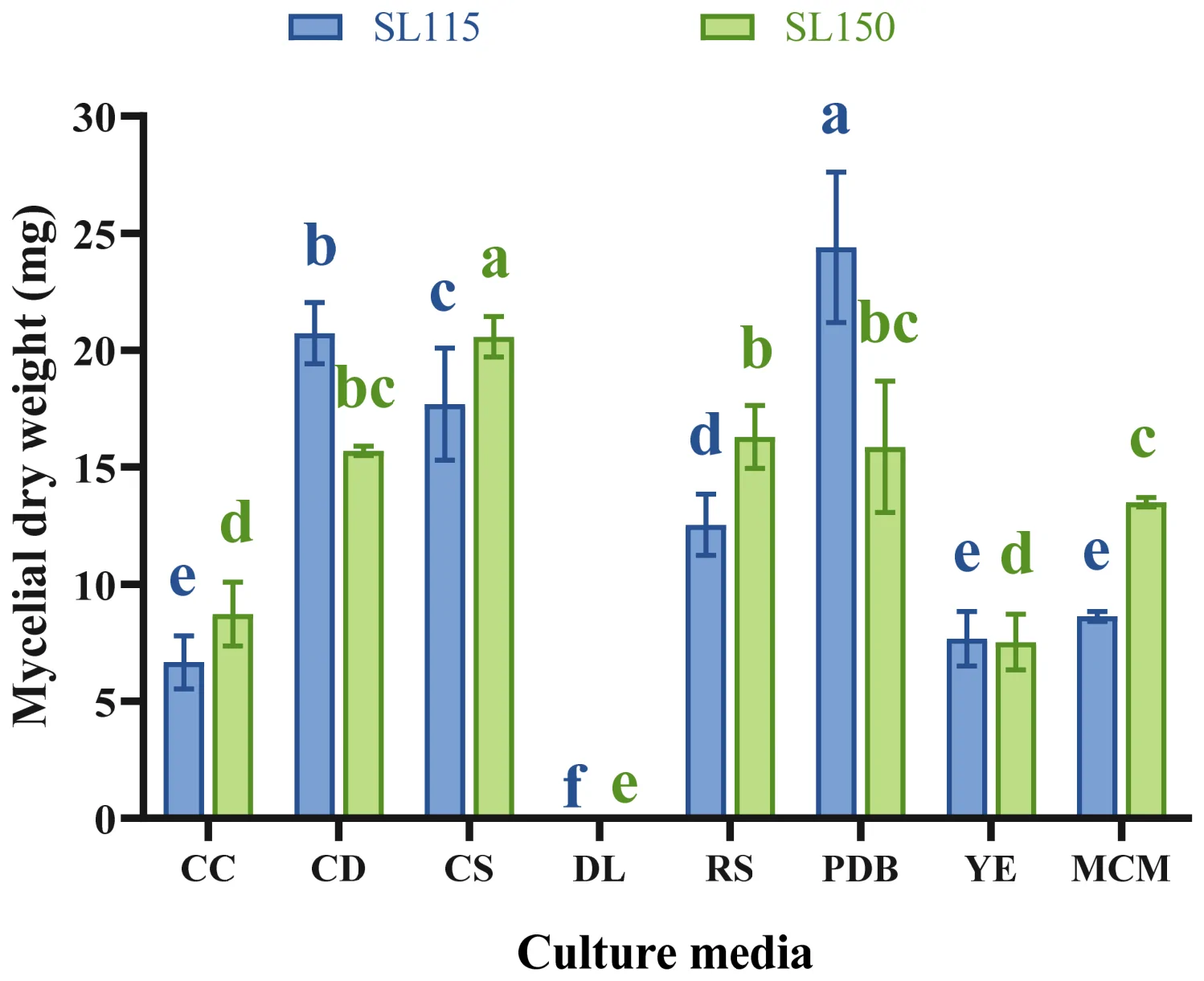
Mycelial dry weight (mg) of two *A. heimuer* strains, SL115 and SL150, on eight different culture media. Data are presented as means ± standard deviation (SD) of five replicates. Different letters above bars indicate statistically significant differences among substrates within each *A. heimuer* strain (*p* ≤ 0.05), according to Duncan’s multiple-range test. Note: CC: corn cob; CD: corn dust; CS: corn stalk; DL: *Dipterocarpus* leaves; RS: rice straw; PDB: potato dextrose broth; YE: yeast extract; MCM: mushroom complete media.

The liquid spawn media were prepared using eight culture media, namely CC, CD, CS, DL, RS, PDB, YE, and MCM. The mycelial dry weights of the two *A. heimuer* strains cultivated in each medium are presented in Figure 6. The highest mycelial dry weight for isolate SL115 was observed on PDB medium (approximately 25 mg), which was significantly higher than that obtained on all other media tested. This result aligns with Jo et al. [45], who reported that potato dextrose (PD)-based media provide suitable conditions for the mycelial growth of *A. auricula-judae*. Following PDB, CD and CS supported moderate mycelial growth, with dry weights of approximately 21 and 18 mg, respectively. In contrast, no mycelial growth was observed on DL medium. For isolate SL150, the highest mycelial dry weight was obtained from CS-based medium, with approximately 20 mg, followed by RS, PDB and CD media, which supported moderate levels of growth. Similarly to SL115, no mycelial growth was detected in DL medium for SL150.

The variation in mycelial growth responses among different agricultural-waste-derived media highlights the influence of nutrient composition on fungal biomass production. A previous study has also demonstrated that *Auricularia* species can efficiently utilize lignocellulosic and agro-industrial residues for growth. For example, Walker et al. [19] reported that *A. cornea* exhibited favorable growth on rice bran sucrose agar (RSA).

#### 3.3.2. Grain Spawn Inoculum

**Figure 7:**
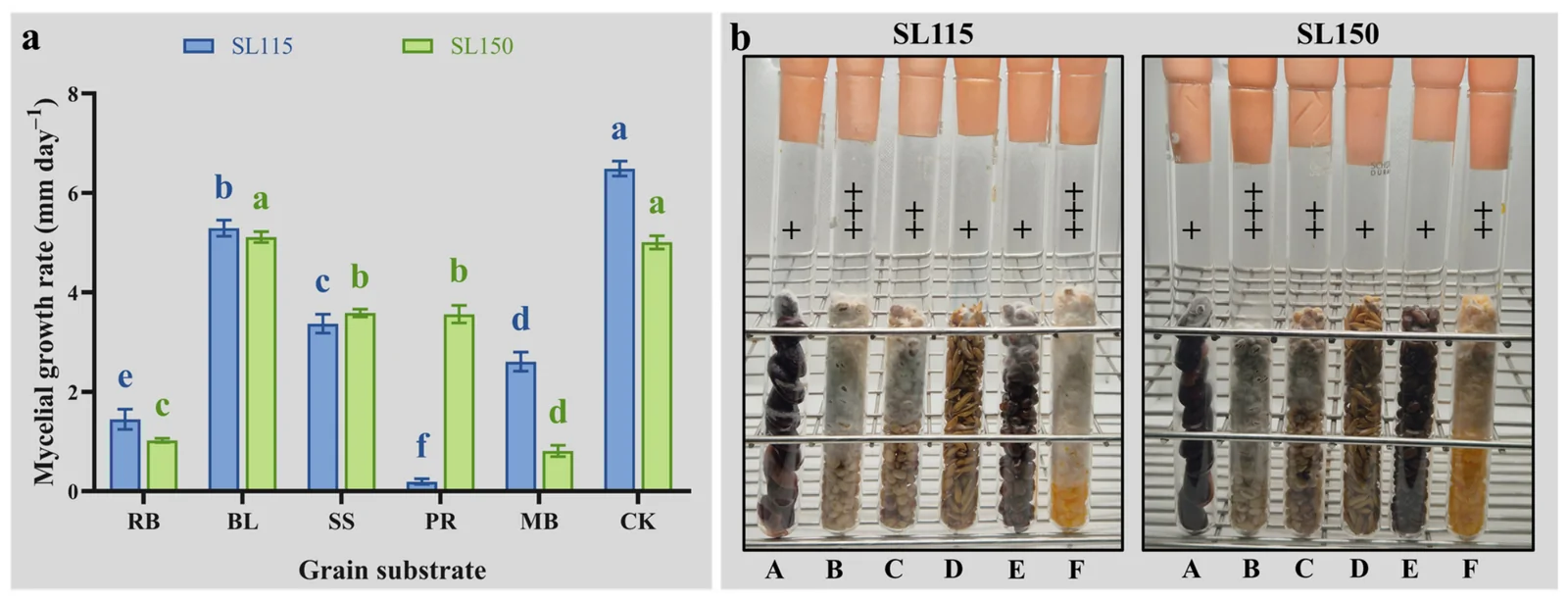
(**a**) Mycelial growth rates (mm day^−1^) of *A. heimuer* strains SL115 and SL150 on six grain substrates (RB: red bean; MB: mung bean; CK: corn kernel; SS: sorghum seed; PR: paddy rice; and BL: barley), after 14 days of incubation. Data are presented as means ± standard deviation (SD) of five replicates. Different letters above bars indicate statistically significant differences among substrates within each strain (*p* ≤ 0.05), according to Duncan’s multiple-range test. (**b**) The appearance of mycelial colonization on grain substrates (A: RB; B: BL; C: SS; D: PR; E: MB; and F: CK). The “+” symbols indicate mycelial density as follows: thick (+++), thin (++), and very thin (+).

The effects of six grain-based substrates, including RB, MB, CK, SS, PR, and BL, were evaluated on the mycelial growth rates of two *A. heimuer* strains (SL115 and SL150). The results indicated that both strains exhibited significant differences in growth performance among the tested grain substrates (Figure 7). Among the evaluated grain substrates, CK was found to be the most suitable for both strains, supporting the highest mycelial growth rates of approximately 6.5 mm day^−1^ for SL115 and 5.0 mm day^−1^ for SL150 (Figure 7a), accompanied by dense colonization [thick mycelia (+++)] in SL115 and thin mycelia (++) in SL150. Barley also supported rapid mycelial growth in both strains, with growth rates of approximately 5.3 and 5.1 mm day^−1^ for SL115 and SL150, respectively, along with dense mycelial colonization (+++ in both strains) (Figure 7b).

The results of this study demonstrated that grain substrate type significantly influences mycelial growth and colonization of *A. heimuer*, consistent with previous reports in cultivated edible mushrooms, where the nutritional composition of spawn substrate plays a critical role in determining mycelial growth [68]. The optimal performance of corn kernels observed in the present study is consistent with the findings of Aguilar et al. [69], where the authors reported that cracked corn seed was the most suitable spawning material for *A. polytricha*, producing vigorous mycelial growth and highly dense mycelia. This may be attributed to the high carbohydrate, protein, vitamin, and mineral contents of corn, which consistently support rapid mycelial colonization in *Auricularia* species [70]. Similar findings have also been reported in other edible mushrooms, where corn-based grains supported the highest mycelial growth rates and the earliest spawn development in *Pleurotus pulmonarius*, *P. tuberregium*, and *Hypsizygus ulmarius* [71].

Barley (BL) also supported rapid mycelial growth in *A. heimuer* strains, which may be associated with its balanced nutrient composition, high water absorption capacity, and retention potential [67,72]. Similar results have been reported in *Pleurotus* species, where barley-based substrates enhanced mycelial extension and spawn quality. The larger grain size provides greater air space properties to barley that may also contribute to faster mycelial colonization [73].

Sorghum seed (SS), which is commonly used as a commercial grain spawn substrate, resulted in moderate mycelial growth in both strains, with a mycelial growth rate of approximately 3.4–3.6 mm day^−1^ and thin mycelial density (++). Mung bean (MB) and RB showed lower growth rates (approximately 0.8–2.6 mm day^−1^), with very thin mycelial density (+). In contrast, PR was the least suitable substrate, particularly for SL115, showing extremely low growth (0.2 mm day^−1^) and sparse mycelial development (+). These findings differ from previous studies on *A. polytricha* and *A. cornea*, which reported that sorghum, millet or paddy rice grains were the most favorable substrates, resulting in the highest mycelial growth rates and the thickest mycelial development [14,19,33,74]. Overall, the results demonstrated that CK and BL are the most effective substrates for grain spawn inoculum, promoting rapid mycelial growth and dense colonization in both *A. heimuer* strains, whereas RB, MB, and particularly PR were less suitable for mycelial growth.

#### 3.3.3. Solid Spawn Inoculum

**Figure 8:**
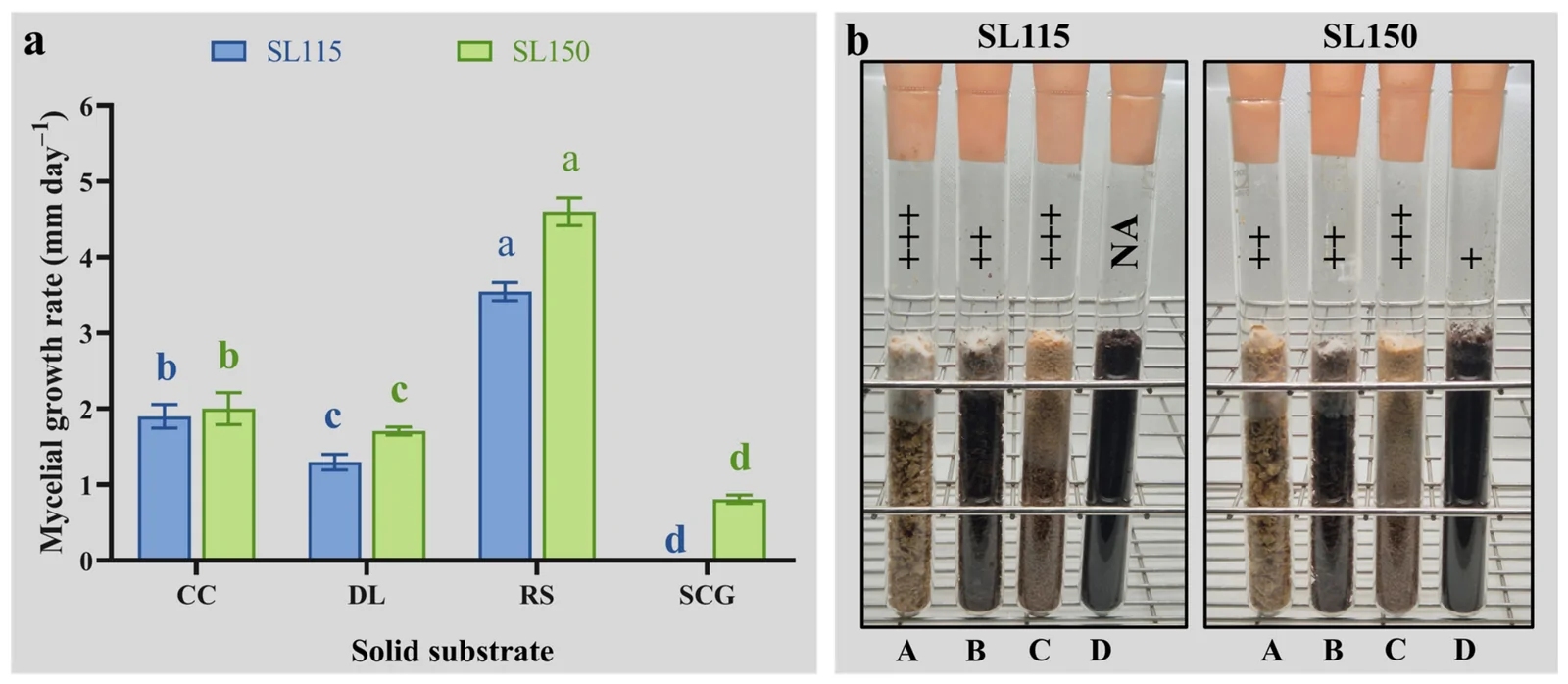
(**a**) Mycelial growth rates (mm day^−1^) of *A. heimuer* strains SL115 and SL150 on four solid substrates (CC: corn cob; DL: *Dipterocarpus* leaves; RS: rubber sawdust; SCG: spent coffee ground), after 14 days of incubation. Data are presented as means ± standard deviation (SD) of five replicates. Different letters above bars indicate statistically significant differences among substrates within each strain (*p* ≤ 0.05), according to Duncan’s multiple-range test. (**b**) The appearance of mycelial colonization on grain substrates (A: CC; B: DL; C: RS; and D: SCG). The “+” symbols indicate mycelial density as follows: thick (+++), thin (++), and very thin (+). N/A = not applicable.

For solid spawn inoculum, four substrates, namely CC, DL, RS, and SCG, were evaluated. Significant differences (*p* ≤ 0.05) in mycelial growth rate were observed among substrates for both isolates (Figure 8). Lignocellulosic materials are widely used as substrates for mushroom cultivation because cellulose and hemicellulose serve as major carbon sources for fungal growth, and variations in substrate composition can directly influence mushroom productivity and quality [75]. In addition to chemical composition, physical properties of the substrate, such as particle size, porosity, moisture retention, and aeration, can also affect mycelial colonization by influencing water availability and oxygen diffusion within the substrate [76]. After 14 days of incubation, RS was found to be the most suitable substrate for both strains. *Auricularia heimuer* SL115 showed the highest mycelial growth rate on RS, measuring approximately 3.5 mm day^−1^, while SL150 exhibited higher growth on the same substrate, at approximately 4.7 mm day^−1^, which was the highest value recorded among all treatments (Figure 8a). This improved performance was also confirmed by the dense mycelial development (+++) observed on RS cultures for both isolates (Figure 8b, tube C).

These results are consistent with previous studies reporting that sawdust is one of the most commonly used substrates for mushroom cultivation, including *Auricularia* species, because of its suitability for supporting mycelial growth and fruiting body production [4,19,69,74]. The favorable performance of RS may be associated not only with its lignocellulosic composition but also with its physical properties, which may provide suitable moisture retention and aeration for mycelial development. Corn cob and DL substrate supported moderate mycelial growth for both strains. In contrast, the use of SCG resulted in the lowest mycelial growth rates; in particular, no growth was observed for SL115. The poor performance of SCG may be associated with the presence of relatively high levels of phenolic compounds, including tannins and chlorogenic acid, as well as caffeine, present in spent coffee ground. These compounds have been reported to interfere with fungal enzyme activity and restrict mycelial colonization during the early growth stage [77]. Similar inhibitory effects of coffee waste have also been reported in *P. salmoneostramineus*, *P. eryngii*, and *P. ostreatus* cultivated in mixed substrates containing high proportions of coffee residues [77]. In addition, *Dipterocarpus* leaf litter contains phenolic compounds, tannins, and sesquiterpenes associated with antimicrobial and cytotoxic activities, which may suppress microbial degradation and consequently reduce fungal growth efficiency [78].

#### 3.3.4. Stick Spawn Inoculum

**Figure 9:**
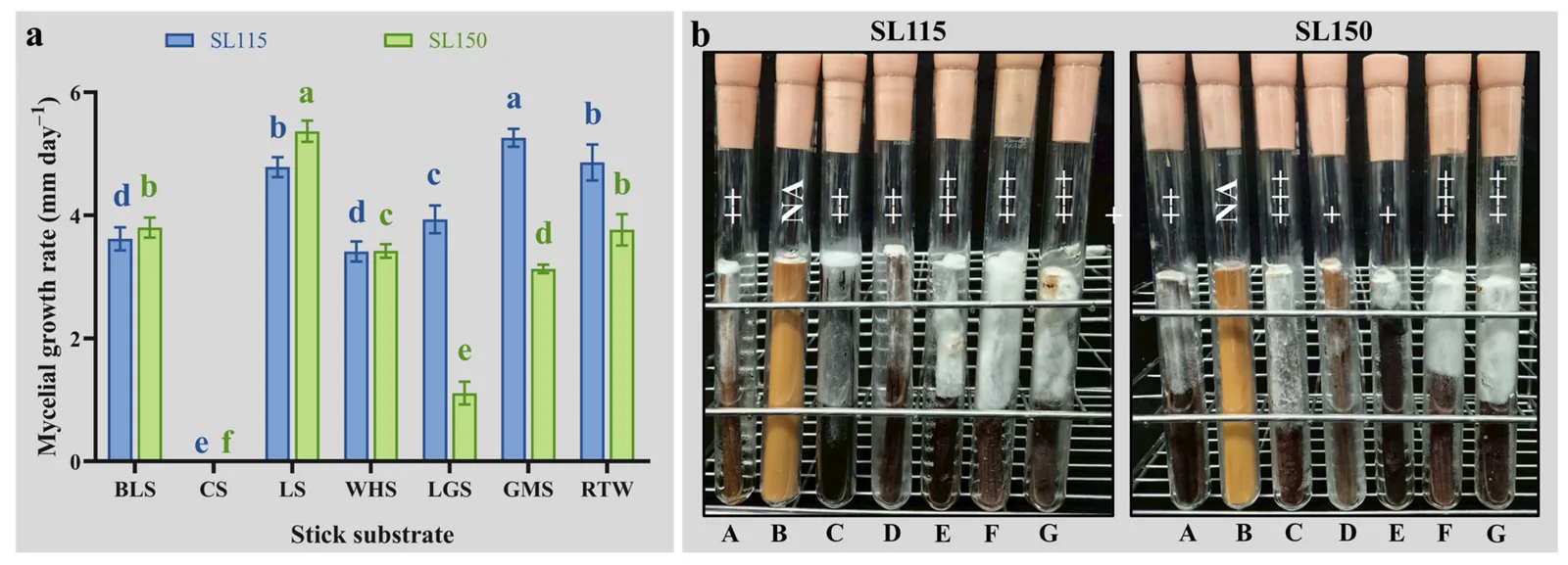
(**a**) Mycelial growth rates (mm day^−1^) of *A. heimuer* strains SL115 and SL150 on seven stick substrates (BLS: banana leaf sheath; CS: corn stalk; LS: lotus stalk; WHS: water hyacinth stalk; LGS: longan stalk; GMS: giant mimosa stalk; RTW: rubber tree wood), after 14 days of incubation. Data are presented as means ± standard deviation (SD) of five replicates. Different letters above bars indicate statistically significant differences among substrates within each strain (*p* ≤ 0.05), according to Duncan’s multiple-range test. (**b**) The appearance of mycelial colonization on grain substrates (A: BLS; B: CS; C: LS; D: WHS; E: LGS; F: GMS; and G: RTW). The “+” symbols indicate mycelial density as follows: thick (+++), thin (++), and very thin (+). N/A = not applicable.

Stick spawn has increasingly been developed as an alternative inoculum type for the cultivation of various mushroom species. This type of spawn is commonly prepared using lignocellulosic materials, including wood sticks, corn stalks, corn cobs, and other plant-based solid substrates. Previous studies have demonstrated that stick spawn can influence mycelial growth and improve BE in mushroom production [79]. Furthermore, lignocellulosic waste, including banana leaf midribs, corn stalks, and broadleaf tree wood, has been investigated as support materials for spawn production and enhancement of fungal biomass development in several edible mushroom species [35,80,81]. In the present study, seven lignocellulosic substrates, including BLS, CS, LS, WHS, LGS, GMS, RTW, were tested for suitability as stick spawn substrates for the mycelial growth of *A. heimuer*. The results demonstrated that the growth performance of the two *A. heimuer* strains varied considerably depending on the substrate type used (Figure 9).

For strain SL115, the most favorable substrate was RTW, which supported the highest mycelial growth rate at approximately 5.2 mm day^−1^ (Figure 9a) and produced thick mycelial density (Figure 9b, Tube F). A different growth pattern was observed for strain SL150. The highest mycelial growth rate was recorded on LS, at approximately 5.3 mm day^−1^ with thick mycelial density (+++), followed by RTW, at approximately 3.8 mm day^−1^, which also produced thick mycelial density. Similarly to strain SL115, CS did not support mycelial colonization in strain SL150. The inability of both *A. heimuer* strains to grow on CS suggests that this material may possess unsuitable physical or chemical properties for fungal growth. This was followed by GMS, which exhibited a growth rate of approximately 4.9 mm day^−1^. In contrast, CS was found to be unsuitable as a substrate for mycelial growth, as no colonization was observed.

These findings are consistent with those reported by Xayyavong et al. [35], who demonstrated that GMS and RTW stick spawn consistently promoted superior mycelial extension and denser mycelial growth of several edible mushrooms, such as *Auricularia polytricha*, *Pleurotus* spp., *Lentinus* spp., *Ganoderma lucidum*, and *Schizophyllum commune*. This effect may be attributed to the favorable lignocellulosic composition of these substrates, as such materials typically contain 35–55% cellulose, 15–40% hemicellulose, and 7–30% lignin, which support efficient enzymatic degradation by wood-decaying fungi [35,82]. Collectively, stick spawn prepared from GMS, RTW, and LS can effectively support the mycelial growth of *A. heimuer*. These results indicate that the type of substrate used in stick spawn preparation plays a significant role in influencing fungal mycelial growth. The significant differences observed in this study are consistent with previous reports by Xayyavong et al. [35] and Desisa et al. [83], who indicated that substrate composition, along with physicochemical properties, nutrient availability, and moisture content, influences the colonization efficiency and metabolic activity of mushroom mycelia.

### 3.4. Mycelial Growth Performance and Contamination Rate of Mushroom Spawn

The mycelial growth performance and contamination rate of mushroom spawn varied markedly among treatments, reflecting strong effects of fungal strain, species, and substrate formulation (Table 2). Overall, complete substrate colonization ranged from 36.40 ± 4.17 to 50.40 ± 4.01 days, with significant differences among treatments. The fastest colonization was observed in T1 (*A. cornea* TMCC No. 8 on 100% S), while the slowest growth occurred in T8 (*A. heimuer* SL150 on 100% S). In contrast, *A. heimuer* SL115 grown on 100% S (T3) failed to complete colonization due to severe contamination (86.67%), indicating strong susceptibility under this substrate condition. When alternative substrate formulations were applied, *A. heimuer* showed improved performance, particularly under mixed lignocellulosic substrates. Treatments incorporating sugarcane leaf combinations mostly supported more stable colonization, although growth rates still varied significantly. Notably, T11 (SL150 with 50S + 50SL) and T9 (SL150 with 50S + 50CS) exhibited relatively efficient colonization (42.60 ± 1.35 and 43.50 ± 4.33 days, respectively), indicating improved adaptability under mixed substrate conditions.

Contamination rate also showed pronounced variation, ranging from 0.00% to 33.33% in most successful treatments. The lowest contamination was recorded in T9 (0.00%), followed by T7, T10, and T12 (6.67%), suggesting that certain substrate combinations create more favorable conditions that both enhance mycelial growth and suppress microbial contamination during spawn development. In contrast, higher contamination levels were observed in several pure sawdust-based treatments, particularly for *A. heimuer*, highlighting the limitations of single-substrate approaches. Overall, these results suggest that *A. heimuer* exhibits variable mycelial growth performance depending on strain selection and substrate composition, with mixed biomass residues providing more favorable conditions than single-formulation substrates. The findings also indicate that substrate optimization plays a crucial role in reducing contamination risk and improving spawn quality. Importantly, this study provides a new record of *A. heimuer* in Thailand, demonstrating its potential for cultivation when appropriate substrate formulations are applied to provide stable and efficient mycelial colonization.

The observed differences in mycelial colonization and contamination among treatments demonstrate that both fungal strain and substrate composition are critical determinants of successful spawn production [19,84]. The superior performance of *A. cornea* TMCC No. 8 on 100% sawdust confirms its broad adaptability to conventional wood-based substrates, whereas the poor performance of *A. heimuer*, particularly strain SL115, indicates that this species exhibits more specific nutritional and environmental requirements during spawn development. The severe contamination and incomplete colonization observed in the 100% sawdust treatment suggest that sawdust alone may not provide an optimal physicochemical environment for *A. heimuer*, thereby increasing its susceptibility to competitive microorganisms. In contrast, mixed substrates containing corn stalks and teak leaves significantly improved colonization efficiency while reducing contamination, indicating that these lignocellulosic materials likely provide a more balanced nutrient composition, improved aeration, and enhanced moisture-holding capacity that favor vigorous mycelial establishment. Similar findings have been reported in previous studies, where supplementing wood substrates with agricultural residues enhanced mycelial growth by increasing substrate porosity and the availability of readily degradable nutrients [13,23,85]. The exceptionally low contamination observed in the 50% sawdust + 50% corn stalk treatment further suggests that an appropriate substrate formulation may simultaneously support mycelial establishment and reduce opportunities for contaminant establishment. Nevertheless, spawn colonization, contamination, and fruiting performance may react differently to substrate formulation and strain [86]. Importantly, contamination rate, mycelial colonization, and yield were not necessarily correlated with substrate type alone. In this study, rapid colonization did not always correspond to low contamination, as shown by T11 and T9. This suggests that substrate composition may interact with strain adaptability to influence mycelial growth and competitive ability against contaminants. Similarly, Liang et al. [21] reported that the substrate supporting the fastest mycelial growth of *A*. *polytricha* did not produce the highest BE, indicating that mycelial colonization alone may not predict subsequent yield. Therefore, substrate suitability should be evaluated collectively based on colonization, contamination, strain adaptability, and fruiting performance. These findings highlight the importance of selecting both suitable fungal strains and optimized substrate formulations for business-related spawn production. Moreover, the successful colonization of *A. heimuer* SL150 on mixed agricultural residues demonstrates that locally available lignocellulosic wastes can effectively support spawn development, providing a practical and sustainable foundation for the future cultivation and commercialization of this newly recorded edible mushroom species in Thailand.

### 3.5. Mushroom Yield and Biological Efficiency Across Different Substrate Formulations

The cultivation performance of *A. heimuer* was evaluated across different substrate formulations and compared with standard strains of *A. cornea*. Overall, the results demonstrated that substrate composition significantly influenced mushroom yield, number of flushes, and BE (Table 3). Among the reference treatments, *A. cornea* SL167 (T2) exhibited the highest productivity, with a fresh weight of 225.77 ± 48.69 g per bag, 7.60 ± 1.35 flushes, and a BE of 133.79 ± 26.69%, significantly outperforming all other treatments. In contrast, *A. heimuer* SL115 grown on 100% common sawdust (T3) showed no measurable data due to severe contamination, indicating poor adaptability under this substrate condition.

**Figure 10:**
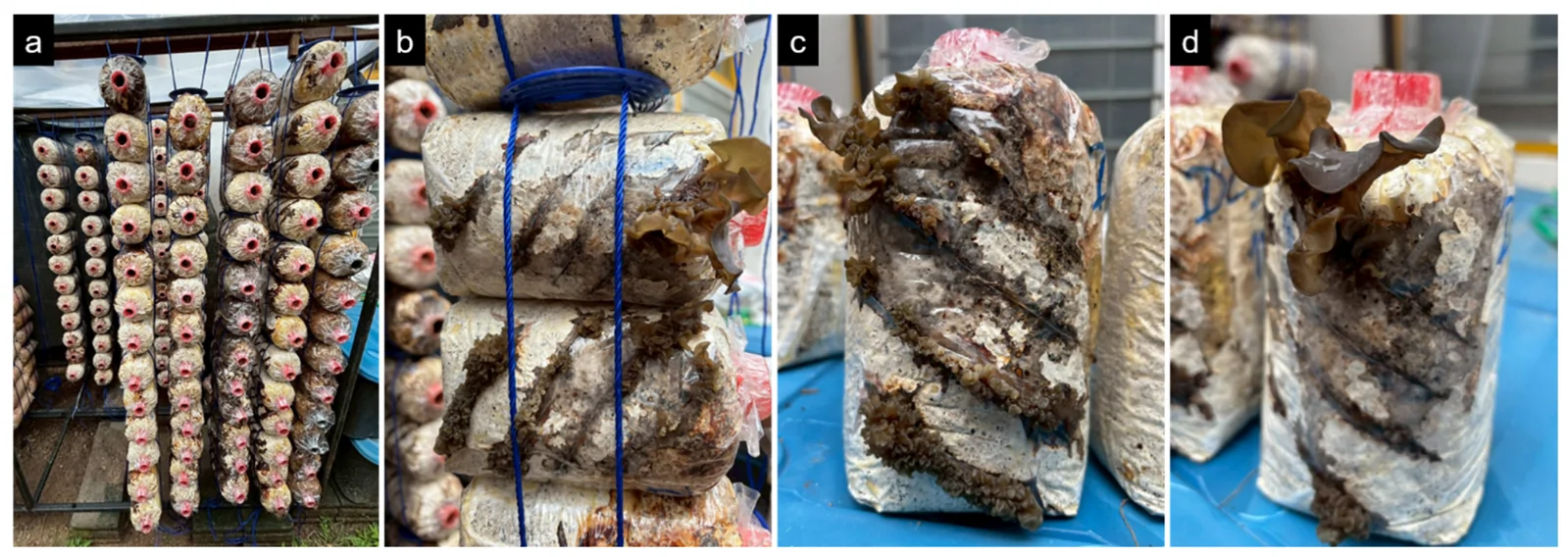
Cultivation and developmental stages of *Auricularia heimuer* (**a**), including primordia formation (**b**), development of young fruiting bodies (**c**), and mature fruiting bodies (**d**), observed after bag opening under substrate cultivation.

For *A. heimuer* cultivation (Figure 10), performance varied markedly depending on substrate formation. In general, supplementation of sawdust with biomass residues improved both yield and BE compared to the 100% sawdust-based formulation (T8), which showed the lowest BE (8.92 ± 2.86%). Among the amended substrates, the combination of sawdust with durian leaves (50S + 50DL; T10) produced the highest yield for *A. heimuer* SL150, reaching 43.86 ± 12.89 g per bag, 6.10 ± 1.45 flushes, and a BE of 37.59 ± 12.08%. Similarly high performance was observed in the sawdust–teak leaf mixture (50S + 50TL; T12), which also achieved a relatively high BE of 35.83 ± 16.50%. In contrast, formulations containing corn stalk with sawdust mixtures (T4 and T9) resulted in moderate productivity, while treatments with sugarcane leaf or TL-based mixtures (T6, T7, and T11) mostly yielded lower outputs and fewer flushes. These results suggest that the physical structure and nutrient availability of the substrate play a crucial role in mycelial development and fruiting body formation.

Notably, although *A. heimuer* showed lower overall productivity than *A. cornea*, its ability to produce acceptable yields under certain biomass-residue-based substrates highlights its potential adaptability to locally available materials. The findings indicate that optimized substrate formulation is essential to enhance cultivation efficiency, particularly for newly introduced or regionally adapted strains such as *A. heimuer* in Thailand.

The significant differences in mushroom yield and BE among substrate formulations indicate that substrate composition is a key determinant of the cultivation performance of *A. heimuer* [13]. In contrast, the higher rate of complete substrate colonization observed in the sawdust–*Dipterocarpus* leaf (50S + 50DL) and sawdust–teak leaf (50S + 50TL) formulations suggests that these mixtures may provide a more favorable balance of lignocellulosic components, physical porosity, aeration, moisture retention, C/N ratio, and nutrient availability, while potentially enhancing enzymatic degradation capacity. These substrate characteristics may have contributed to more efficient mycelial colonization and sustained fruiting. This finding is consistent with previous research on *A. polytricha*, in which a sawdust-based substrate supplemented with different proportions of stalks from three grass species was evaluated. Liang et al. [21] reported that sawdust supplemented with *Pennisetum purpureum* stalks affected the total colonization time, showing that incorporating alternative lignocellulosic materials into sawdust-based substrates can influence substrate colonization and cultivation performance. Similar findings have been reported for *Auricularia* spp., where substrate physicochemical properties, including cellulose, hemicellulose, lignin content, and C:N ratio, strongly influence fruiting body development and BE [13,20,44]. Although *A. heimuer* produced lower yields than the commercial *A. cornea* strain, its satisfactory performance on selected agro-residue mixtures demonstrates its capacity to utilize locally available agricultural wastes effectively. These findings highlight the importance of substrate optimization for newly introduced strains and support the use of agricultural by-products as sustainable cultivation substrates for expanding *A. heimuer* production in Thailand.

### 3.6. Nutritional Composition of Harvested Mushrooms

**Figure 11:**
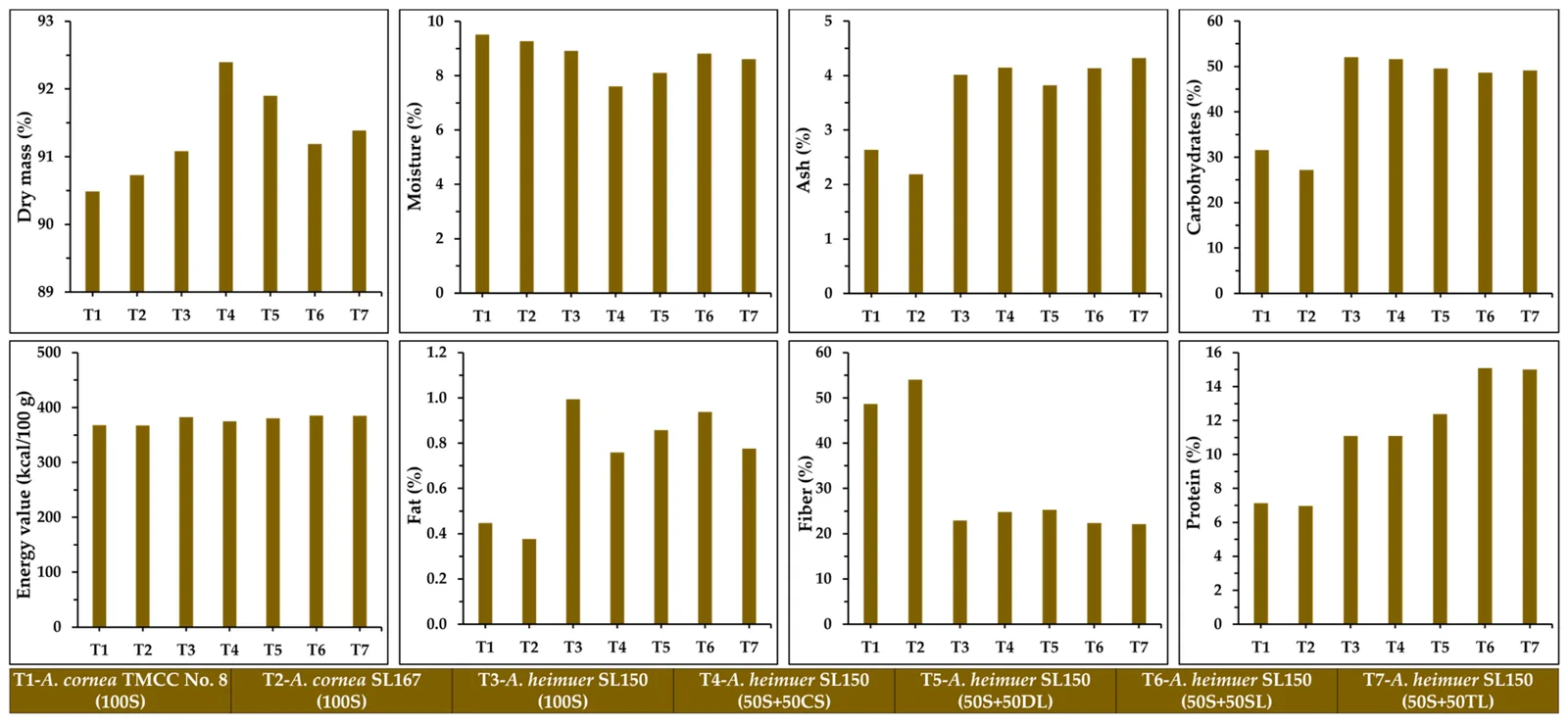
Proximate composition (%) and energy values (kcal/100 g) of dried *A. heimuer* SL 150 fruiting bodies compared with *A. cornea* TMCC No. 8 and SL167 cultivated on different substrate formulations. Values are presented as means for each treatment. Note: The differences shown do not represent statistical conclusions; they reflect only numerical variation among samples. Abbreviation: S = sawdust; DL = Dipterocarpus leaves; SL = sugarcane leaves; TL = teak leaves.

The proximate composition and energy values of dried fruiting bodies of *A. heimuer* SL150, compared with *A. cornea* TMCC No. 8 and SL167, revealed clear variations influenced by both species and substrate formulations (Figure 11). Overall, samples exhibited typical characteristics of edible mushrooms, with relatively high carbohydrate and dietary fiber contents, moderate protein levels, and low-fat content. These traits confirm their potential as functional foods with favorable nutritional profiles.

Among the treatments, *A. heimuer* SL150 cultivated on optimized mixed substrates mostly showed better nutritional composition compared with the comparison strains. In particular, protein and ash contents tended to be higher in SL150 fruiting bodies, indicating enhanced nutrient assimilation from the substrate. Carbohydrate content remained the dominant fraction across all treatments, contributing substantially to the caloric value. Meanwhile, crude fat levels were consistently low in all samples, reflecting the inherent lipid-poor nature of basidiomycete mushrooms. Dietary fiber content also showed noticeable variation, with SL150-based treatments exhibiting relatively acceptable levels in several substrate combinations, although lower than the reference strains.

Comparatively, *A. cornea* TMCC No. 8 and SL167 showed slightly lower or more stable nutrient profiles across treatments. This indicates that *A. heimuer* SL150 may have a stronger response to substrate optimization in terms of nutritional enhancement. The observed differences highlight the important role of substrate composition in regulating biochemical accumulation in fruiting bodies. In addition, the variation among the 50S + 50CS, 50S + 50DL, 50S + 50SL, and 50S + 50TL formulations suggest species-specific utilization efficiency of lignocellulosic materials. Such effects are likely driven by differences in enzymatic degradation capacity and nutrient uptake efficiency. Overall, these results demonstrate that *A. heimuer* SL150 is a promising candidate for cultivation in Thailand with greater nutritional quality under optimized substrate conditions.

The nutritional composition of *A. heimuer* SL150 fruiting bodies demonstrates that substrate formulation plays a pivotal role in determining the biochemical quality of edible mushrooms [87]. The higher protein and ash contents observed in SL150 cultivated on optimized mixed substrates indicate enhanced nutrient acquisition and mineral translocation resulting from the more efficient degradation of lignocellulosic materials. Similar findings have been reported for edible mushrooms, where substrate composition directly influences carbon and nitrogen availability, thereby regulating protein biosynthesis and mineral accumulation during fruiting body development [13,17,20]. The consistently low-fat content and high carbohydrate levels across all treatments are characteristic of *Auricularia* species, further supporting their reputation as nutritious, low-calorie functional foods [17]. Variations in dietary fiber content suggest that lignocellulose-rich substrates may stimulate the accumulation of structural polysaccharides within fungal cell walls, while the slightly higher energy values observed in mushrooms cultivated on mixed agricultural residues (e.g., 50S-based formulations) may be attributed to improved carbon availability and greater metabolic efficiency during fungal growth. These findings further indicate that mixed substrates can modify fungal metabolism by enhancing substrate accessibility and carbon utilization efficiency [13]. Moreover, the superior nutritional performance of *A. heimuer* SL150 compared with the *A. cornea* reference strains suggests species-specific differences in lignocellulose-degrading enzyme systems and nutrient assimilation capacities. Collectively, these results demonstrate that optimized mixed substrates not only improve cultivation performance but also enhance the nutritional quality of harvested mushrooms, highlighting the potential of locally available agricultural residues for producing high-value functional foods while promoting sustainable mushroom production in Thailand.

## 4. Conclusions

This study reports *Auricularia heimuer* as a newly recorded species in Thailand based on morphological characteristics and phylogenetic analyses based on ITS and nLSU regions, thereby extending its known distribution from temperate East Asia to the tropical dipterocarp forests of Thailand and revealing a novel host association with members of the Dipterocarpaceae. Mycelial growth was significantly influenced by culture medium, temperature, pH, and nitrogen source, with strain-specific optima generally occurring at approximately 30 °C, under acidic to neutral pH conditions, and in media supplemented with organic nitrogen sources. In addition, substrate suitability varied among the different spawn types evaluated. Cultivation trials demonstrated that supplementation of the sawdust substrate with *Dipterocarpus* or teak leaves significantly enhanced mushroom yield and BE compared with the standard substrate. Furthermore, the harvested fruiting bodies exhibited favorable nutritional composition. Collectively, these findings establish *A. heimuer* as a promising edible mushroom for commercial cultivation in Thailand and demonstrate the potential for converting locally available lignocellulosic residues into value-added agricultural products. Further multilocus phylogenetic analyses, together with genomic, transcriptomic, and metabolomic studies, are needed to determine whether genetic or physiological adaptations contribute to differences in temperature and substrate responses between tropical and temperate *A. heimuer* strains. Additionally, large-scale cultivation trials are recommended to further optimize production efficiency and facilitate the development of standardized commercially industrial cultivation systems.

## Figures and Tables

**Table 1:** Mycelial growth and biomass yield of three strains of *A. heimuer* grown on culture media supplemented with different nitrogen sources after seven days.

Nitrogen Source	Growth Rate (mm day^−1^)			Biomass Dry Weight (mg plate^−1^)		
	SL115	SL150	SL160	SL115	SL150	SL160
KNO_3_	6.40 ± 0.21 ^c^	6.96 ± 0.18 ^d^	6.94 ± 0.15 ^c^	6.62 ± 2.60 ^c^	6.60 ± 1.11 ^e^	3.82 ± 1.00 ^d^
NH_4_Cl	4.96 ± 0.05 ^d^	4.62 ± 0.15 ^f^	3.82 ± 0.13 ^f^	2.46 ± 0.43 ^de^	18.62 ± 3.17 ^c^	18.98± 4.15 ^bc^
(NH_4_)_2_SO_4_	5.40 ± 0.00 ^d^	6.32 ± 0.11 ^e^	4.10 ± 0.07 ^e^	3.24 ± 0.68 ^d^	7.40 ± 0.98 ^e^	3.44 ± 1.83 ^d^
NaNO_3_	0.00 ± 0.00 ^e^	0.00 ± 0.00 ^g^	0.00 ± 0.00 ^g^	0.00 ± 0.00 ^e^	0.00 ± 0.00 ^f^	0.00 ± 0.00 ^d^
Malt extract	6.12 ± 0.04 ^c^	8.22 ± 0.08 ^c^	8.02 ± 0.23 ^a^	6.00 ± 0.08 ^c^	11.72 ± 1.45 ^d^	15.46 ± 1.60 ^c^
Peptone	7.82 ± 0.87 ^b^	8.88 ± 0.25 ^a^	7.28 ± 0.29 ^b^	28.66 ± 0.81 ^a^	24.70 ± 2.25 ^b^	24.96 ± 3.59 ^a^
Tryptone	8.62 ± 0.59 ^a^	6.48 ± 0.04 ^e^	5.42 ± 0.23 ^d^	29.38 ± 3.11 ^a^	27.86 ± 4.80 ^ab^	21.40 ±5.86 ^ab^
Yeast extract	8.18 ± 0.48 ^ab^	8.48± 0.30 ^b^	7.08 ± 0.15 ^bc^	21.30 ± 4.71 ^b^	29.05 ± 4.54 ^a^	21.68 ± 2.62 ^ab^
Urea	0.00 ± 0.00 ^e^	0.00 ± 0.00 ^g^	0.00 ± 0.00 ^g^	0.00 ± 0.00 ^e^	0.00 ± 0.00 ^f^	0.00 ± 0.00 ^d^

**Note:** KNO_3_ = potassium nitrate; NH_4_Cl = ammonium chloride; (NH_4_)_2_SO_4_ = ammonium sulfate; NaNO_3_ = sodium nitrate. Each value is represented as mean ± SD (n = 5). Different letters within the same column indicate significant differences (*p* ≤ 0.05) according to Duncan’s multiple-range test.

**Table 2:** Mycelial colonization and contamination rates of mushroom spawn produced on different substrate formulations.

Treatments	Completed Substrate Colonization (Days)	Contamination Rate (%)
T1-*A. cornea* TMCC No. 8 (100S)	36.40 ± 4.17 ^e^	13.33
T2-*A. cornea* SL167 (100S)	45.50 ± 2.88 ^bcd^	20.00
T3-*A. heimuer* SL115 (100S)	ns	86.67
T4-*A. heimuer* SL115 (50S + 50CS)	44.50 ± 1.90 ^bcd^	33.33
T5-*A. heimuer* SL115(50S + 50DL)	47.30 ± 4.81 ^b^	26.67
T6-*A. heimuer* SL115 (50S + 50SL)	45.10 ± 2.88 ^bcd^	33.33
T7-*A. heimuer* SL115 (50S + 50TL)	43.80 ± 2.78 ^cd^	6.67
T8-*A. heimuer* SL150 (100S)	50.40 ± 4.0 ^a^	33.33
T9-*A. heimuer* SL150 (50S + 50CS)	43.50 ± 4.33 ^cd^	0.00
T10-*A. heimuer* SL150 (50S + 50DL)	46.00 ± 2.71 ^bc^	6.67
T11-*A. heimuer* SL150 (50S + 50SL)	42.60 ± 1.35 ^d^	20.00
T12-*A. heimuer* SL150 (50S + 50TL)	43.30 ± 1.16 ^cd^	6.67

Note: Values are expressed as means ± standard deviation (SD). Different letters within the same column indicate significant differences according to Duncan’s multiple-range test (*p* ≤ 0.05). Contamination rate (%) was determined from 15 replicate spawn bags. Ns = no data due to high-level contamination. Abbreviation: S = sawdust; DL = Dipterocarpus leaves; SL = sugarcane leaves; TL = teak leaves.

**Table 3:** Overall mushroom yield, number of flushes, and biological efficiency of each substrate formulation across the cultivation period.

Treatments	Fresh Weight per Bag (g)	Number of Flushes	BE (%)
T1-*A. cornea* TMCC No. 8 (100S)	164.02 ± 42.33 ^b^	6.00 ±1.25 ^b^	104.94 ± 19.89 ^b^
T2-*A. cornea* SL167 (100S)	225.77 ± 48.69 ^a^	7.60 ± 1.35 ^a^	133.79 ± 26.69 ^a^
T3-*A. heimuer* SL115 (100S)	ns	ns	ns
T4-*A. heimuer* SL115 (50S + 50CS)	21.55 ± 6.78 ^de^	3.50 ± 1.35 ^cde^	25.80 ±9.24 ^cd^
T5-*A. heimuer* SL115(50S + 50DL)	26.18 ±13.43 ^cde^	3.70 ± 1.16 ^cd^	26.52 ± 13.85 ^cd^
T6-*A. heimuer* SL115 (50S + 50SL)	16.30 ± 7.49 ^def^	3.50 ± 1.43 ^cde^	20.98 ± 10.73 ^de^
T7-*A. heimuer* SL115 (50S + 50TL)	17.15 ± 7.32 ^def^	2.10 ± 0.88 ^e^	19.48 ± 8.16 ^de^
T8-*A. heimuer* SL150 (100S)	14.01 ± 4.21 ^ef^	2.80 ± 0.79 ^de^	8.92 ± 2.86 ^ef^
T9-*A. heimuer* SL150 (50S + 50CS)	17.23 ± 6.93 ^def^	4.51 ± 3.20 ^c^	13.41 ± 5.40 ^de^
T10-*A. heimuer* SL150 (50S + 50DL)	43.86 ± 12.89 ^c^	6.10 ± 1.45 ^b^	37.59 ± 12.08 ^c^
T11-*A. heimuer* SL150 (50S + 50SL)	24.29 ± 10.76 ^cde^	3.40 ± 1.26 ^cde^	17.74 ± 8.49 ^de^
T12-*A. heimuer* SL150 (50S + 50TL)	37.35 ± 17.78 ^cd^	3.90 ± 1.73 ^cd^	35.83 ± 16.50 ^c^

Note: Values are expressed as means ± standard deviation (SD). Different letters within the same column indicate significant differences according to Duncan’s multiple-range test (*p* ≤ 0.05). Ns indicates missing data due to high-level contamination that prevented measurement. Abbreviation: S = sawdust; DL = *Dipterocarpus* leaves; SL = sugarcane leaves; TL = teak leaves.

## Data Availability

Data are contained within the article.

## Supplementary Materials

The following supporting information can be downloaded at: https://www.mdpi.com/article/10.3390/life16101647/s1, Table S1: Information of sequences used for phylogenetic analyses of *Auricularia* in the present study; Table S2: A morphological comparison of *Auricularia heimuer* and *A. minutissima* (the new collection from this study is indicated in bold).
