# Supplementary material for: A New Record of Auricularia heimuer in Thailand: Optimal Conditions for Mycelial Growth, Inoculum Production, Cultivation Performance and a Favorable Nutritional Profile
Source: Life (Basel). 2026 Sep 30;16(10):1647. doi: 10.3390/life16101647 (PMC13705923; doi:10.3390/life16101647)
Supplement: Supplementary file 1 [file life-16-01647-s001.zip › life-4463505-supplementary.pdf]

Supplementary Table S1. Information of sequences used for phylogenetic analyses of *Auricularia* in the present study.

Species name	Strains code	Country	GenBank accession number	
			ITS	nLSU
Auricularia africana	Ryvarden 44929^T	Uganda	MH213350	MZ669897
A. africana	T3	Kenya	MH213349	MZ669918
A. americana	Cui 11509	China	KT152094	KT152110
A. americana	Dai 13461	China	KM396763	KM396821
A. angiospermarum	Cui 12360^T	USA	KT152097	KT152113
A. angiospermarum	HHB 11037	USA	KT152098	KT152114
A. asiatica	BBH 895^T	Thailand	KX621160	N/A
A. asiatica	Dai 15285	China	KX022009	KX022040
A. auricula-judae	Dai 13210	France	KM396769	KM396824
A. auricula-judae	JT 04	UK	KT152099	KT152115
A. australiana	HN 3213	Australia	MZ647504	MZ669921
A. australiana	HT 190^T	Australia	MZ647503	MZ669920
A. brasiliana	AN-MA 42^T	Brazil	KP729275	KP729293
A. brasiliana	RSC 359	Brazil	KP729276	KP729294
A. camposii	URM 76905^T	Brazil	MH213351	MH213395
A. camposii	URM 83464	Brazil	MH213352	MH213396
A. conferta	Dai 18825^T	Australia	MZ647500	MZ669901
A. conferta	Dai 18826	Australia	MZ647505	N/A
A. cornea	Dai 15336	China	KX022014	KX022045
A. cornea	MFLU162110	Thailand	KX621141	N/A
A. delicata	P 14E^T	Cameroon	MH213364	MZ669933
A. cornea	YG-Dr1	Germany	MH213353	MH213397
A. fibrillifera	F 234519^T	Papua New Guinea	KP765610	KP765624
A. fibrillifera	MFLU 24-0195	Thailand	PP935774	PP935773
A. fuscosuccinea	Dai 17422	Brazil	MH213367	MH213408
A. fuscosuccinea	FP-102573-SP	USA	KX022027	KX022058
A. heimuer	AFM 21	Japan	AB615232	N/A
A. heimuer	Dai 13765^T	China	KM396793	KM396844
A. heimuer	Dai 2291	China	KM396785	KM396838
A. heimuer	LE 296433	Russia	KJ698423	N/A
A. heimuer	SL115	Thailand	PZ586918	N/A
A. heimuer	SL150	Thailand	PZ586919	N/A
A. heimuer	SL160	Thailand	PZ586920	PZ586923
A. lateralis	Dai 15670^T	China	KX022022	KX022053
A. lateralis	Dai 16416	China	KX022023	KX022054
A. mesenterica	BRNM 648573	Czech Republic	KP729279	KP729297
A. mesenterica	Haikonen 11208	UK	KP729287	KP729305
A. minutissima	Dai 14881^T	China	KT152104	KT152120

Species name	Strains code	Country	GenBank accession number	
			ITS	nLSU
A. minutissima	Dai 15453	China	MH213371	MH213412
A. minutissima	Dai 15455	China	KX022030	KX022061
A. nigricans	Ahti 36234	Costa Rica	KM396802	KM396850
A. nigricans	TJY-93-242	USA	KM396803	KM396851
A. novozealandica	PDD 81195	New Zealand	KX022033	KX022064
A. novozealandica	PDD 83897^{T}	New Zealand	KX022034	KX022065
A. orientalis	Dai 14875^{T}	China	KP729270	KP729288
A. orientalis	Dai 1831	China	KP729271	KP729289
A. pilosa	JMH 45	Tanzania	KM267731	KM267738
A. pilosa	LWZ 20190421-7^{T}	Ethiopia	MZ647506	N/A
A. pusio	AK 547	Australia	MH213374	MH213414
A. pusio	Smith 18	Zambia	MH213375	MZ669926
A. scissa	Ahti 49388	Dominican Republic	KM396805	KM396853
A. scissa	TFB 11193^{T}	Dominican Republic	JX065160	N/A
A. sinodelicata	CMUB40117	Thailand	PX601577	PX601576
A. sinodelicata	Cui 8596	China	MH213376	MH213415
A. sinodelicata	Dai 13926^{T}	China	MH213379	MZ669909
A. srilankensis	Dai 19522^{T}	Sri Lanka	MZ647501	MZ669912
A. srilankensis	Dai 19575	Sri Lanka	MZ647502	MZ669913
A. subglabra	JV 1808/125	Guiana	MZ618941	MZ669911
A. subglabra	Wu 08	Brazil	MH213384	MZ669928
A. submesenterica	Dai 15449	China	KX022039	KX022070
A. submesenterica	Dai 15450^{T}	China	MH213386	MH213420
A. thailandica	MFLU130410^{T}	Thailand	KR336693	N/A
A. thailandica	MFLU130411	China	KR336694	N/A
A. tibetica	Cui 12267^{T}	China	KT152106	KT152122
A. tibetica	Cui 12337	China	KT152108	KT152124
A. tremellosa	AJS 1304	Mexico	JX065158	N/A
A. tremellosa	Dai 17415	Brazil	MH213390	MH213424
A. villosula	LE 296422^{T}	Russia	NR137873	N/A
A. villosula	MFLU 16-2126	Thailand	KX621165	N/A
Exidia subsaccharina	BJFC 036778^{T}	France	NR198312	NG243790
Heteroradulum australiense	20180515-26^{T}	Australia	MZ325256	MZ310426

Note: Ex-type strains are marked as "T" and newly described strains are indicated in red. The unavailable sequences are indicated as "N/A".

Supplementary Table S2. A morphological comparison of *Auricularia heimuer* and *A. minutissima* (the new collection from this study is indicated in bold).

Characteristics	*Auricularia heimuer* Wu et al. [6, 8]	***Auricularia heimuer* (this study)**	*Auricularia minutissima* Wu et al. [6, 9]
Basidiomata	Up to 12 cm	**Up to 13 cm**	Up to 4 cm
Abhymenial hair	50–150 × 4–6.5 µm	**50–150 × 3–6.5 µm**	50–105 × 5–6 µm
Basidia	40–67 × 3–6.5 µm	**36–67 × 3.0–6.5 µm**	46–67 × 5–7 µm
Basidiospores	11–13 × 4–5 µm	**11–14 × 4.5–6 µm**	12–16 × 5–7 µm
Medulla	present	**present**	present
Host	angiosperm	**angiosperm**	angiosperm
Locality	China, Japan and Russia	**Thailand**	China and Russia
